# Long‐Lived Charge‐Transfer State Induced by Spin‐Orbit Charge Transfer Intersystem Crossing (SOCT‐ISC) in a Compact Spiro Electron Donor/Acceptor Dyad

**DOI:** 10.1002/anie.202003560

**Published:** 2020-05-08

**Authors:** Dongyi Liu, Ahmed M. El‐Zohry, Maria Taddei, Clemens Matt, Laura Bussotti, Zhijia Wang, Jianzhang Zhao, Omar F. Mohammed, Mariangela Di Donato, Stefan Weber

**Affiliations:** ^1^ State Key Laboratory of Fine Chemicals School of Chemical Engineering Dalian University of Technology 2 Ling Gong Road Dalian 116024 China; ^2^ Division of Physical Sciences and Engineering King Abdullah University of Science and Technology (KAUST) Thuwal 23955-6900 Kingdom of Saudi Arabia; ^3^ LENS (European Laboratory for Non-Linear Spectroscopy) via N. Carrara 1 50019 Sesto Fiorentino (FI) Italy; ^4^ Institute of Physical Chemistry Albert-Ludwigs-Universität Freiburg Albertstrasse 21 79104 Freiburg Germany; ^5^ ICCOM-CNR via Madonna del Piano 10 50019 Sesto Fiorentino (FI) Italy

**Keywords:** charge transfer, electron spin control, intersystem crossing, time-resolved EPR, triplet state

## Abstract

We prepared conceptually novel, fully rigid, spiro compact electron donor (Rhodamine B, lactam form, RB)/acceptor (naphthalimide; NI) orthogonal dyad to attain the long‐lived triplet charge‐transfer (^3^CT) state, based on the electron spin control using spin‐orbit charge transfer intersystem crossing (SOCT‐ISC). Transient absorption (TA) spectra indicate the first charge separation (CS) takes place within 2.5 ps, subsequent SOCT‐ISC takes 8 ns to produce the ^3^NI* state. Then the slow secondary CS (125 ns) gives the long‐lived ^3^CT state (0.94 μs in deaerated n‐hexane) with high energy level (ca. 2.12 eV). The cascade photophysical processes of the dyad upon photoexcitation are summarized as ^1^NI*→^1^CT→^3^NI*→^3^CT. With time‐resolved electron paramagnetic resonance (TREPR) spectra, an EEEAAA electron‐spin polarization pattern was observed for the naphthalimide‐localized triplet state. Our spiro compact dyad structure and the electron spin‐control approach is different to previous methods for which invoking transition‐metal coordination or chromophores with intrinsic ISC ability is mandatory.

## Graphical abstract

**A rigid, simple, spiro** compact electron donor/acceptor (D/A) dyad shows a long‐lived triplet charge transfer (^3^CT) state (0.94 μs) with a high energy level (ca. 2.12 eV), based on a new electron spin‐control method using spin‐orbit charge transfer intersystem crossing (SOCT‐ISC) (^1^NI*→^1^CT→^3^NI*→^3^CT; NI=naphthalimide), without heavy atoms or chromophores with intrinsic intersystem crossing (ISC) ability.

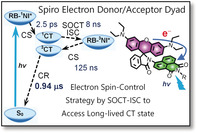

## Introduction

Mimicking the natural photosynthetic reaction centers with the aim to attain a charge transfer (CT) state with high energy and long lifetime has attracted much attention.^1^, ^2^, ^3^, ^4^, ^5^, ^6^ In photosynthesis, a cascade of electron‐transfer processes takes place in the reaction center upon photoexcitation of the light‐harvesting antenna, leading to the formation of a final CT state at approximately 0.5 eV, that lives up to a few seconds.5a, 7, 8 Molecular systems that exhibit such long‐lived CT states are of fundamental importance for artificial photosynthesis, photovoltaics, molecular electronics/photonics and photochemistry studies.^9^, ^10^, ^11^, ^12^, ^13^ Controlling electron‐transfer is also pivotal for fluorescent molecular sensors^14^, ^15^, ^16^ and charge recombination (CR) induced persistent luminescence.^17^ Various donor(D)/acceptor(A) polyads mimicking natural photosynthesis have been reported in the past years to generate long‐lived CT states.^18^, ^19^ To this scope, it is necessary to attain large electronic coupling and efficient charge separation (CS) between adjacent electron D and A in each step, and weak electronic coupling between primary electron D and terminal electron A (a large distance between the two units is required) to retard CR process. However, very often stepwise electron transfer produces CT states with rather low energy.

The non‐adiabatic CT rate *k*_CT_ depends on Δ*G*_CT_, and *k*_CT_ should decrease as the driving force (−Δ*G*_CT_) increases in the so‐called Marcus inverted region, that is, higher CT state energy usually leads to long‐lived CT states.^3^, 5a, 20 However, it is not always reliable to obtain a long‐lived ^1^CT state based on the Marcus inverted region effect with the classic Marcus equations, because the electron transfer (or CR) rate constants may be much larger than those theoretically predicted in the Marcus inverted region, due to the quantum tunneling effect.5a

The lifetime of a ^1^CT state is usually shorter than that of a ^3^CT state because ^1^CT→S_0_ is a spin‐allowed transition. ^3^CT state usually has a longer lifetime than the ^1^CT state, enabling the so‐called electron spin control effect. Radical pair intersystem crossing (RP ISC) mechanism can be used as an electron spin control method, in which the probability of ^1^CT→^3^CT transition increases, thus prolonging the lifetime of the CT states. RP ISC accounts on hyperfine interactions (HFI) to facilitate ISC in electron D/A dyads/polyads with an extremely small electronic coupling (<0.1 cm^−1^, so that rigid and long bridges are required to connect electron D and A).5a, 21 However, a small energy gap between ^1^CT and ^3^CT states may lead to backward transition of ^3^CT→^1^CT, and the ^1^CT→^3^CT transition (several nanoseconds) is usually slower than ^1^CT→S_0_ decay.5a These issues cause short‐lived CT states.

A possible alternative to attain long‐lived CT states is to use the electron spin control approach to initiate the CS with a triplet state precursor.^22^, ^23^, ^24^ One possibility is to use a transition‐metal coordination framework to attach the electron D and A because the coordination center has usually an ultrafast ISC, through a ^1^MLCT→^3^MLCT→^3^CT process (ML=metal to ligand).^22^, ^24^ For instance, a long‐lived CT state (ca. 1 μs) was observed in a N^N Pt^II^ bis(acetylide) complex and the formation of a spin‐correlated radical pair (SCRP) was confirmed by TREPR.^22^ These molecular systems are synthetically demanding, because the π‐conjugation frameworks of electron D and A must be isolated from the coordination center, to reduce the electronic coupling between electron D and A and avoid the shortening of the ^3^CT state lifetime (heavy‐atom effect).5a, 6, 22 Another strategy is based on the intrinsic ISC ability of the electron D or A (e.g. anthraquinone (Aq)‐D linked systems) to establish ^1^LE→^3^LE→^3^CT (LE: locally excited state), without any heavy atoms.5b, 25 In a reported cyclopeptide‐derived spiro electron D/A system, the ^3^CT lifetime is 3.35 μs in THF.5b The molecular structure is quite complicated and this method is restricted to a limited number of chromophores exhibiting intrinsic ISC with high yield. Thus, it is still a major challenge to construct simple, compact electron D/A compact dyads to access long‐lived ^3^CT states.

Herein, we report a new electron spin control approach, based on the spin‐orbit charge transfer ISC (SOCT‐ISC) mechanism, to obtain a long‐lived ^3^CT state (0.94 μs, at room temperature),^22^, ^26^, ^27^, ^28^ using a novel, simple, fully rigid, spiro electron D (lactam form Rhodamine B, RB)/electron A (naphthalimide, NI) compact dyad to induce orthogonal orientation and a fully rigid connection between RB and NI units. In our case, SOCT‐ISC will generate ^3^NI* state (^1^CT→^3^NI* transition). ^3^NI* is the precursor to form a long‐lived ^3^CT state via charge separation, achieving electron spin control. As demonstrated by steady‐state and time‐resolved optical and magnetic resonance spectroscopies, the proposed photophysical processes of **RB‐NI** dyad are ^1^NI*→^1^CT→^3^NI*→^3^CT. Our strategy will be useful for designing simple, compact electron D/A dyads showing long‐lived ^3^CT state with high energy.

## Results and Discussion

### Molecular Structure Design Rationale

**Figure 1:**
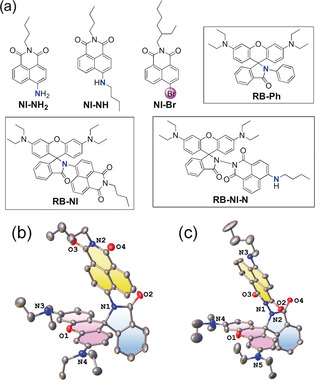
a) Molecular structures of rhodamine–naphthalimide derivatives and reference compounds. ORTEP view of the molecular structures determined with single‐crystal X‐ray diffraction of the compounds of b) **RB‐NI** and c) **RB‐NI‐N**; hydrogen atoms and solvents are omitted for clarity. The xanthene moieties are highlighted in pink and NI in yellow. Thermal ellipsoids are set at 50 % probability. CCDC numbers are given in the Supporting Information.

Inspired by high energy ^3^LE state of NI (ca. 2.20 eV),^6^ we selected NI moiety as electron A for formation of a low‐lying ^3^CT state. Spiro rhodamine with high energy excited states will not be involved in dyad's photophysical processes. The structures of the dyads and reference compounds are shown in Figure 1 a. The highly constrained conformation of **RB**‐**NI** will reduce π‐conjugation between the N atom and NI moiety (the π‐conjugation frameworks are separated by three σ‐bonds), which is crucial for NI moiety to maintain its high T_1_ energy and strong electron withdrawing ability. Spiro rhodamine is linked with the NI moiety at the amide‐position in the reference dyad **RB**‐**NI**‐**N**, note for the dyad **RB‐NI** the link is at the 4‐amino position of the NI moiety. For **RB‐NI‐N**, less conformational restriction is expected (refer to the potential energy curves in Supporting Information, Figure S14f). Hence, π‐conjugation between the N atom and the NI's π‐framework is significant for **RB‐NI‐N**, which will decrease the energy of ^3^LE below that of the CT state. The reduced electron withdrawal ability of the NI moiety in **RB‐NI‐N** will promote the energy of the CT state, as compared to that of **RB‐NI** (Supporting Information, Table S5). Recently the aggregation induced luminescence property of **RB‐NI** was reported, but the charge separation was not studied.28b

The use of a short rigid linker will reduce the reorganization energy *λ* of electron transfer, which is beneficial for fast CS and for attaining long‐lived CT state.5a, 7 However, this also makes the electron exchange energy (*J*) large, increasing the ^1^CT–^3^CT energy gap (2*J*), and inhibiting ^3^CT→^1^CT process (by HFI). For conventional electron D/A dyads, the very small *J* value makes the ^3^CT/^1^CT energy very similar, promoting the reverse ^3^CT→^1^CS which drains ^3^CT.^29^ In our method, SOCT‐ISC is supposed to render ^1^CT→^3^NI* process fast, and the close proximity of NI and the RB units to increase the *J* value, thus suppressing the undesired backward ^3^CT→^3^NI*→^1^CT processes.

Single crystals of **RB‐NI** and **RB‐NI‐N** were obtained by slow evaporation of the solutions of the dyads (CH_2_Cl_2_/*n*‐hexane for **RB‐NI**, CHCl_3_/*n*‐hexane for **RB‐NI‐N**). The molecular structures of the dyads were thus characterized by single‐crystal X‐ray diffraction (XRD; Figure 1, Supporting Information, Table S1 and S2). **RB‐NI** crystallized in the triclinic crystal system in the $P\bar{1}$ space group with two molecules in a unit cell and **RB‐NI‐N** crystallized in the monoclinic crystal system in the *P*2_1_/*n* space group. The dihedral angle between xanthene and NI moieties is 70.8° in **RB‐NI** and 66.7° in **RB‐NI‐N** (Figure S13b), the former being more perpendicular than predicted by density functional theory (DFT) at the B3LYP/6‐31G(d) level (60.4° for **RB‐NI**, 67.6° for **RB‐NI‐N**, Figure S14a and b).^30^ These geometries are beneficial for SOCT‐ISC.^26^, ^27^, ^31^, ^32^, ^33^, ^34^

### UV/Vis Absorption and Luminescence Spectra

**Figure 2:**
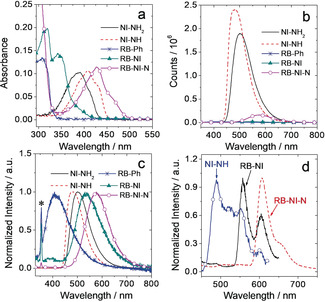
a) UV/Vis absorption spectra of the compounds in *n*‐hexane, *c*=1.0×10^−5^ m. b) Fluorescence emission spectra (optically matched solutions were used, *A*=0.113, *λ*_ex_=320 nm) and c) the corresponding normalized fluorescence emission spectra of the compounds in *n*‐hexane, 20 °C. The asterisk in (c) indicate the Raman scattering peak of the solvent at 354 nm. d) Normalized phosphorescence spectra of the compounds **RB‐NI** (*λ*_ex_=340 nm), **NI‐NH** (*λ*_ex_=340 nm) and **RB‐NI‐N** (*λ*_ex_=425 nm) at 77 K, in mixed solvents of *n*‐hexane/iodoethane (4:1, v/v), *c*=5.0×10^−5^ m.

The steady‐state UV/Vis absorption spectra of the compounds were studied (Figure 2 a). For **RB‐NI** with its nearly orthogonal conformation, we expect weak electronic coupling between electron D and A. The absorption band of **RB‐NI** (342 nm) is blue‐shifted as compared to that of **NI‐NH_2_** (390 nm) and **RB‐NI‐N** (427 nm). We attribute this blue shift to the electron‐deficient nature of the N atom at 4‐position of NI moiety, which is connected to the carbonyl group (amide). The molecular conformation also plays a role in this respect, because constrained molecular geometry reduces the π‐conjugation of the N atom with NI moiety in **RB‐NI**.28a

Compared to reference compounds, the fluorescence of NI moiety in **RB‐NI** is strongly quenched (Figure 2 b and Table 1). The residual fluorescence of NI moiety in **RB‐NI** is centered at 400 nm (Figure 2 b,c), which is shorter in wavelength and much weaker than that of **NI‐NH_2_**. This blue‐shifted fluorescence emission is an indication of the limited π‐conjugation between the amino N atom and NI moiety. We propose that quenching of the NI fluorescence in **RB‐NI** is due to photo‐induced electron transfer between RB and NI moieties, which is supported by cyclic voltammetry studies (Figure S16, Table S5) and the analysis of frontier molecular orbitals of **RB**‐**NI** (Figure S14c). The weak broad CT emission band of **RB**‐**NI** and **RB‐NI‐N** are at around 545 nm and 578 nm (Figure 2 c), respectively. The photophysical parameters of the dyads are listed in Table 1. The fluorescence of **RB‐NI‐N** is stronger (Φ_F_=10.7 %) than that of **RB‐NI** (Φ_F_=1.2 %), but it is much weaker than that of **NI‐NH** (Φ_F_=67.5 %). This result indicates that electron transfer is also possible for **RB‐NI‐N**. Temperature‐dependent luminescence of **RB‐NI** were measured, however, thermal activated delayed fluorescence did not occur (Figure S15).

**Table 1:** Photophysical parameters of the compounds.

Compounds	*λ*_abs_^[a]^ [nm]	*ϵ* ^[b]^	*λ*_F_^[c]^ [nm]	*Φ*_Δ_^[d]^ [%]	*Φ*_F_^[e]^ [%]	*τ*_F_^[f]^ [ns]	*λ*_P_^[g]^ [nm]	*τ*_P_^[h]^ [ms]	*τ*_T_^[i]^
**NI‐NH_2_**	390	1.03	500	^[l]^	67.2	8.6	^[l]^	^[l]^	^[l]^
**NI‐NH**	408	1.06	480	^[l]^	67. 5	7.5	490	20.3	^[l]^
**RB‐Ph**	308	1.35	410	^[l]^	1.2	4.7	^[l]^	^[l]^	^[l]^
**RB‐NI**	319/342	1.99/1.44	545	29.8	1.2	15.7	556/604	20.0	125 ns^[j]^ / 935 ns ^[k]^
**RB‐NI‐N**	427	1.15	578	24.5	10.7	18.4	606	36.3	50.5 μs

[a] UV/Vis absorption maxima in *n*‐hexane, *c*=1.0×10^−5^ m, 25 °C. [b] Molar absorption coefficient at absorption maxima, *ϵ*: 10^4^ m ^−1^ cm^−1^. [c] Maximal fluorescence emission wavelength in *n*‐hexane, absorbance *A*=0.113, 25 °C. [d] Singlet oxygen quantum yield in *n*‐hexane, Ru(bpy)_3_[PF_6_]_2_ was used as standard (*Φ*_Δ_=57 % in ACN).35a [e] Absolute photoluminescence quantum yield in *n*‐hexane, determination error: ±0.1 %, *A*=0.10, 25 °C. [f] Luminescence lifetime in *n*‐hexane, *c*=1.0×10^−5^ m, *λ*_ex_=340 nm. [g] Maximal phosphorescence wavelength in *n*‐hexane/iodoethane (4:1, v/v), *c=*5.0×10^−5^ m, 77 K. [h] Phosphorescence lifetime in *n*‐hexane/iodoethane (4:1, v/v), *c=*5.0×10^−5^ m, 77 K. [i] Triplet state and ^3^CT state lifetimes, determined with nanosecond transient absorption spectroscopy. In *n*‐hexane. [j] Growth kinetics of the transient at 425 nm in the nanosecond transient absorption spectra. [k] Decay kinetics of the transient at 425 nm in the nanosecond transient absorption spectra. [l] Not observed.

Phosphorescence spectra of the dyads at 77 K were measured (Figure 2 d, iodoethane was added to induce external heavy atom effect, which will be helpful on enhance the phosphorescence). The phosphorescence lifetimes are presented in Table 1. The energy levels of the ^3^NI* state of **RB**‐**NI** and **RB**‐**NI**‐**N** can be approximated as 2.21 eV and 2.10 eV, respectively. The energy of the CT state of the dyads can be evaluated based on electrochemical data (Table S5).

### Femtosecond, Subnanosecond, and Nanosecond Transient Absorption Spectra: Charge Separation, Charge Recombination and Intersystem Crossing

To verify the photophysical processes involved in the dyads upon pulsed laser excitation, we measured transient absorption (TA) spectra of **RB‐NI**, **RB‐NI‐N** and reference compounds.

Femtosecond TA (fs TA) spectra of **NI‐Br** in toluene shows that ISC occurs within 30 ps (Figures S19), similarly to what reported for non‐brominated NI, with ISC time constants of 10–20 ps.35b To obtain the absorption of the ^3^NI state and the NI radical anion (NI^−.^), the nanosecond TA (ns TA) spectra of **NI**‐**Br** in the absence and presence of sacrificial electron donor triethylamine(TEA) were recorded (Figure S24). In the absence of TEA, two excited state absorption (ESA) bands at 380 nm and in the range of 400–600 nm are observed for **NI**‐**Br**, assigned to ^3^NI state. The triplet state lifetime in deaerated solution is 30.0 μs (Figure S24b). In the presence of TEA, the ESA bands of the ^3^NI state at 485 nm and 380 nm decay faster (*τ*=6.3 μs, Figure S24d). A new absorption band at 422 nm grows concomitantly (Figure S24c). These spectral changes are attributed to the intermolecular electron transfer, with TEA as sacrificial electron D and **NI**‐**Br** as electron A, which generates NI radical anion (NI^−.^). The apparently longer CS and longer lifetime of NI^−.^ absorption for **NI**‐**Br**/TEA mixture are reasonable, considering that an intermolecular diffusion‐controlled electron transfer occurred.

**Figure 3:**
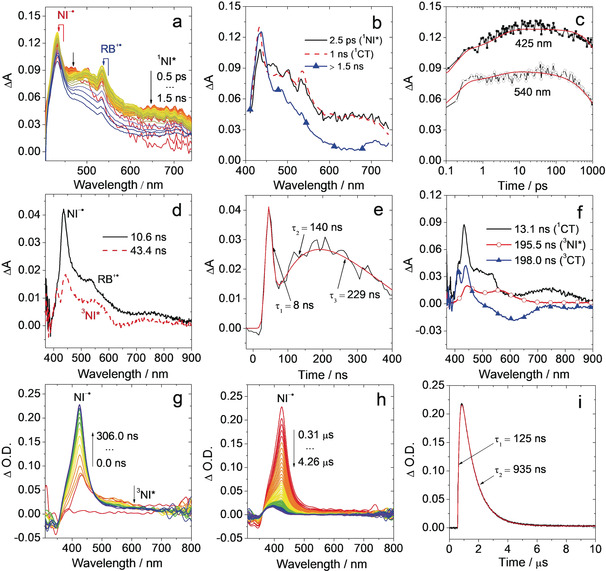
Spectra of **RB‐NI**. a) Femtosecond transient absorption spectra, color code goes from red to blue covering the time interval from 0.5 ps to 1.5 ns, b) EADS obtained from global analysis and c) decay kinetics at 425 nm and 540 nm; spectra were recorded up to 1.5 ns, *λ*_ex_=400 nm, in deaerated toluene. d) Sub‐nanosecond transient absorption spectra, e) decay trace at 434 nm and f) species‐associated difference spectra (SADS) obtained from target analysis. *λ*_ex_=350 nm, in toluene. See text for details. g) Nanosecond transient absorption spectra and h) the subsequent decay of the ESA band at 425 nm at longer delay times. i) Evolution kinetics at 425 nm (derived from (g) and (h)); in deaerated *n*‐hexane, excited with nanosecond pulsed laser, *λ*_ex_=350 nm, *c*=4.0×10^−5^ m, 20 °C.

For **RB‐NI**, the evolution of fs TA spectra is presented in Figure 3 a. With global analysis,^36^ the evolution‐associated difference spectra (EADS) were obtained (Figure 3 b; there are only two species, ^1^NI* and ^1^CT states; the comparison of the fit obtained using two‐component and three‐component models for the TA data of the **RB‐NI** in toluene are shown in Figure S20, demonstrating that a third component significantly improves the fit). Upon pulsed laser excitation at 400 nm, a broad excited state absorption (ESA) band in the range of 600–750 nm is visible, similar to that of **NI‐NH_2_** (Supporting Information, Figures S18a), and assigned to ^1^NI* state. Within 2.5 ps (Figure 3 b), we observe the growth of two sharp peaks at 425 nm and 540 nm. The decay kinetics at 425 nm and 540 nm (Figure 3 c) are similar and do not show complete recovery on the nanosecond timescale, thus implying the possibility of slow ISC; a more accurate estimate of the last time constant (>1.5 ns in Figure 3 b) is presented in Figure 3 d. Based on spectroelectrochemical data (Figure S17) and ns TA spectra of NI^−.^ absorption (Figure S24c), the ESA band at 425 nm can be assigned to NI^−.^ absorption and the RB radical cation (RB^+.^) is at around 540 nm. To better understand the photophysics of **RB‐NI**, fs TA measurements were repeated in acetonitrile (Figures S21). CS in this polar solvent is extremely fast, occurring within less than 1 ps (Figures S21b). CR is also fast, occurring in less than 100 ps, thus implying that the triplet state is not populated.

Sub‐ns TA spectra for **RB‐NI** were recorded (Figure 3 d) to evaluate the kinetics of ISC process. A target analysis with a model of reversible excited‐state processes related to ^1^CT→^3^NI* and ^3^NI*→^3^CT was applied (Figure 3 f).^36^ However, there are no evidence for the reverse processes, for example, ^3^CT→^3^NI*, via target analysis. The initial spectrum shows a sharp ESA band at 425 nm, a moderate band at around 540 nm and a broad, weak ESA band at 700–900 nm, attributed to ^1^CT state (Figure 3 d,f, it is very similar to that obtained on fs TA spectra). The decay at around 425 nm is accompanied with the formation of ^3^NI* state, indicated by the appearance of two ESA bands at 447 nm and 570 nm (red line with circle symbols in Figure 3 f). The blue line with triangle symbols in Figure 3 f shows a sharp ESA band at around 425 nm and a broad negative band at around 600 nm that can be assigned to stimulated CT emission. The decay kinetic at 434 nm (Figure 3 e) shows that the ^1^CT state is formed rapidly upon photoexcitation, then the formation of ^3^NI* state occurs via SOCT‐ISC with timescale approximately 8 ns (^1^CT→^3^NI*).

The ns TA spectra of **RB**‐**NI** recorded upon pulsed laser excitation at 350 nm were measured. We observed the growth of an ESA band at 425 nm, assigned to the NI^−.^ absorption (Figure 3 g),5b, 6, 22 concurrently with the decrease of a broad weak ESA band in the range of 480–650 nm which is attributed to ^3^NI* state of **RB**‐**NI** (Figure 3 g). Thus the formation of the final transient species of **RB**‐**NI** is driven by the ^3^NI precursor (^3^NI*→^3^CT transition) and it is long‐lived (0.94 μs in deaerated *n*‐hexane, Figure 3 i). Its lifetime was ca. 0.16 μs in aerated *n*‐hexane (Figure S22c) and 0.62 μs in deaerated toluene (Figure S23c). In other polar solvents, the lifetime is further shortened (Table S6). No signal was observed in CH_3_CN, in agreement with fs TA spectra (Figures S21). Similarly, in a previously reported cyclopeptide derived electron D/A dyad, the CT state lifetime shows strong solvent polarity dependency.^22^ The decreased CT state lifetime was attributed to the energy gap law,^6^ because the CT state is better stabilized in more polar media. Conversely, the ^3^LE state is usually not sensitive to solvent polarity, and the effect of the electron spin inhibition on the decay kinetics of the T_1_ state is more significant than the energy gap law. Thus, the final transient species observed for **RB**‐**NI** is a ^3^CT state, not a ^3^LE state.

To exclude the possibility of intermolecular electron transfer, which is a diffusion‐controlled process possibly resulting in slow CS/CR kinetics, we used a highly viscous solvent, polydimethylsiloxane (PDMS, viscosity *η*=0.48±0.029 Pa s, much higher than *n*‐hexane, *η*=3.3×10^−4^ Pa s) to study the slow CS process. In this case, a similar kinetic is observed for the growth of NI^−.^ absorption at 425 nm (101 ns). The decay of the band peaked at 425 nm was ca. 0.94 μs (Figure S25c), similar to that in deaerated *n*‐hexane (Figure 3 i). Thus, the slow CS and CR kinetics in deaerated *n*‐hexane are not due to intermolecular electron transfer. Weak electronic coupling between the electron D and A, and a small driving force may result in slow CS and CR.5a, 6, 37 The slow CS of **RB**‐**NI** (125 ns in deaerated *n*‐hexane, Figure 3 i) suggests a weak electronic coupling between the electron D and A. The small CS driving force (^3^NI/^3^CT states energy gap) may also contribute to the slow CS.^6^ Recently, Zn porphyrin unit was connected with rectilinear rigid oligo‐*p*‐xylene bridge with fullerene (C_60_) unit, it was found the efficiency and the kinetics of CS/CR are highly dependent on the electronic coupling magnitude between the donor and acceptor (CT state lifetime: 0.56 μs).37b

**Figure 4:**
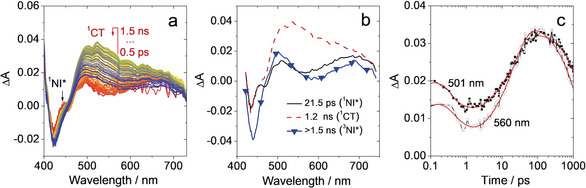
a) Femtosecond transient absorption spectra of **RB‐NI‐N**, color code goes from red to blue covering the time interval from 0.5 ps to1.5 ns, b) EADS of **RB‐NI‐N** obtained from global analysis and c) decay kinetics at 501 nm and 560 nm; in deaerated toluene, 20 °C.

In the fs TA spectra of **RB‐NI‐N**, we observe a broad ESA band with two peaks at about 450–530 nm and 530–750 nm (black line in Figure 4 b), and a ground state bleaching (GSB) band centered at 430 nm. The ESA band intensified substantially within 20 ps, undergoing a change in shape, thus showing a single broad peak at around 550 nm (Figure 4 b). The trace at 560 nm decays faster than that at 501 nm (Figure 4 c), indicating the conversion from the initially excited singlet (^1^NI*) state towards a CT state. The NI^−.^ band is invisible in this case and it may be obscured by the GSB band. Within 1 ns, the ESA band undergoes further evolution, decreasing in intensity and changing again to a double‐peak structure (Figure 4 b). On the same timescale the negative peak also increases, possibly due to the recovery of NI^−.^ signal at a similar spectral position but with opposite sign. The spectrum of the long‐lived component (Figure 4 b, blue line with triangular marks) is assigned to the triplet state of the NI moiety. From global analysis we can thus estimate that the initial charge separation occurs in **RB‐NI‐N** within about 20 ps and the timescale of the charge recombination induced ISC is about 1 ns.^36^

**Figure 5:**
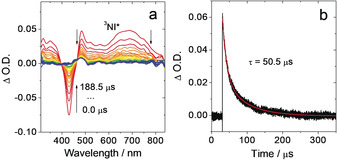
a) Nanosecond transient absorption spectra of **RB‐NI‐N** and b) decay trace of **RB‐NI‐N** at 485 nm. Excited with nanosecond laser at 427 nm, *c=*5.0×10^−5^ m in deaerated *n*‐hexane; 20 °C.

The triplet state formation of **RB‐NI‐N** were also studied with ns TA spectra (Figure 5). A broad ESA band in the range of 450–800 nm (Figure 5 a) is observed, similar to fs TA data of **RB‐NI‐N** (Figure 4). The transient species of **RB‐NI‐N** shows a lifetime of 50.5 μs in deaerated *n*‐hexane. The lifetime is reduced to 0.68 μs in aerated *n*‐hexane (Figure S26). Importantly, the transient lifetime of **RB‐NI‐N** shows no strong solvent polarity dependency. These observations confirm the transient signal of **RB‐NI‐N** is a ^3^LE state, which is different from **RB‐NI**.

### Pulsed Laser Excited Electron Paramagnetic Resonance Spectra (TREPR): Electron Spin Polarization Dynamics

TREPR is a powerful tool for studying the spin multiplicity of the CT states, as well as ^3^LE states; 25c, 38, 39 misinterpretation of data from transient optical spectroscopy on these states may thus be avoided. The ^3^CT and the ^3^LE can be easily distinguished by the zero‐field splitting (ZFS) parameters, *D* and *E*, as well as the specific electron spin polarization (ESP) pattern.^26^, ^40^

**Figure 6:**
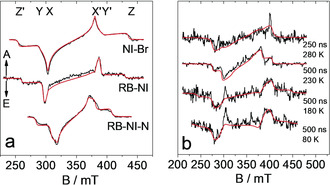
a) TREPR spectra recorded at 80 K in frozen toluene/MeTHF solution, after pulsed laser excitation (355 nm for **NI‐Br** and **RB‐NI**, and 427 nm for **RB‐NI‐N**; the laser pulse length ca. 3–5 ns, 1.5 mJ per pulse; integration time window is 500–700 ns after the laser pulse). The red curves are simulations of the experimental TREPR spectra (black curves). b) TREPR spectra of **RB**‐**NI** in the liquid‐crystal E7 (the glass transition temperature is 210 K) recorded at different temperatures after pulsed laser excitation (355 nm, ca. 3–5 ns, 1.5 mJ per pulse).

To study the CT state and to confirm the ISC mechanism, the TREPR spectra of triplet states of **NI‐Br**, **RB‐NI** and **RB‐NI‐N** recorded in frozen solution at 80 K are presented in Figure 6 a. Spectral simulations of the TREPR data revealed the ZFS parameters, |*D*| and |*E*|, as well as the zero‐field populations, *p*_1_, *p*_2_,and *p*_3_. These can be assigned to *p_x_*, *p_y_* and *p_z_* under the assumption of a disk‐like spin‐density distribution, that is typically applied for planar aromatic systems.^41^ In Figure 6 a, the signs of the ZFS parameters are as follows: *D*>0 and *E*>0 (all simulation parameters are compiled in Table S7). The ZFS parameters *D* and *E* of **RB‐NI** triplet are very similar to those of ^3^NI state in **NI‐Br**, indicating the triplet state is localized on NI moiety of **RB‐NI**. The ESP patterns of **RB‐NI** and **RB‐NI‐N** are EEEAAA (where A denotes enhanced absorption and E denotes emission), the same as that of **NI‐Br** (SO‐ISC). The simulations show that the triplet states in **NI‐Br** and **RB‐NI** have the *p_x_* and *p_y_* sublevels overpopulated; for **RB‐NI‐N**, the populations *p_x_* and *p_y_* are similar, but the *p_x_* sublevel is slightly overpopulated (Table S7).

**Scheme 1:**
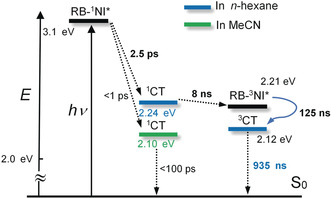
Simplified Jablonski diagram illustrating the photophysical processes involved in **RB‐NI**. ^1^CT energy levels were calculated based on the electrochemical data and TDDFT computations. TDDFT calculations were performed at the B3LYP/6‐31G(d) level by using Gaussian 09W. The triplet excited state energy levels of **RB‐NI** were from the phosphorescence at 77 K.

The centroids of xanthene and NI planes of **RB‐NI** and **RB‐NI‐N** assume distances of 5.104 Å and 5.566 Å (XRD data), respectively. Thus, spin‐spin exchange interactions between the radical anion and the cation of **RB‐NI** and **RB‐NI‐N** will be strong.^6^, ^22^ Only the signal of ^3^NI state was detected in **RB‐NI** and **RB‐NI‐N** samples recorded at 80 K. The lack of any ^3^CT state signal of **RB‐NI** under the conditions used for the TREPR experiments may be due to the increased energy level of CT state in frozen solution at 80 K, and it is consistent with the ns TA spectra results measured at same condition (Figure S28).^25^ In the frozen solution at low temperature, the solvent molecules cannot reorient to stabilize a changed electron‐density distribution of the molecule (lack of effective solvation). As a result, the energy level of the CT state is expected to increase as compared to that at room temperature (note: in toluene solution at room temperature, the ^3^CT state lies only slightly lower than the ^3^LE state, by ca. 0.1 eV. Scheme 1). Thus, the energy of the ^3^CT state may be higher than that of the ^3^LE state in frozen solution, and consequently, the ^3^CT state was not detected by TREPR (Figure 6 a). We exclude the RP ISC mechanism from our TREPR spectral data, because the specific ESP pattern of a triplet state formed via the RP ISC is AEEAAE or EAAEEA, was not observed.

The liquid crystal E7 was used as a solvent for **RB‐NI** to measure TREPR data at more elevated temperature (Figure 6 b, a nematic phase is formed above 210 K). The TREPR signals from **RB‐NI** in liquid‐crystal nematic phase are very noisy, even if measured at low temperature. Therefore, the ZFS parameters can be given only with relatively large uncertainties. At 80 K and 180 K, rather large *D* values around 100 mT are obtained that resemble the ones observed for **NI‐Br**. Hence, under these conditions, the triplet state is rather confined and likely localized on the NI moiety of **RB‐NI**. As in toluene/MeTHF mixture, the population *p_x_* is very high. Above the glass transition temperature in the nematic phase, at 230 and 280 K, a triplet species with a much smaller *D* value approximately 65 mT was observed, comparable to that of **RB‐NI‐N**. This is indicative for stronger spatial delocalization of the triplet wavefunction. Tentatively we assign this triplet species to the rhodamine (RB) moiety of **RB‐NI‐N**. As in the latter molecule, the population *p_x_* and *p_y_* are comparable in size: *p_x_*≈*p_y_*. Within the glassy phase of E7, at 80 and 180 K, an enhanced absorption feature shows up at around 300 mT, that is outside the simulation of a spin‐polarized triplet species (Figure 6 b, lower two traces). This resonance could in principle arise from a CT state, however, due to the poor signal‐to‐noise ratio, we refrain from a definite assignment.

The energy diagrams of **RB‐NI** and **RB‐NI‐N** are presented in Scheme 1 and Scheme S2. For **RB‐NI**, the CS (2.5 ps) process with ^1^NI* as precursor is exoergic (Δ*G*_CS_°=−0.85 eV, Table S5), which leads to the formation of ^1^CT state. The subsequent SOCT‐ISC (8 ns) produces the ^3^NI* state. Then a secondary, slow CS (125 ns in deaerated *n*‐hexane) occurs with ^3^NI* state as precursor. The final low‐lying state is a ^3^CT state (0.94 μs, in deaerated *n*‐hexane). The energy level of ^3^CT of RB‐NI in *n*‐hexane is approximately 2.12 eV, which is much higher than that occurring in the natural photosynthetic centers (ca. 0.5 eV).^7^ The CT state energy of **RB‐NI** is high enough to drive catalytic processes.2a Note although the ^3^CT and ^3^LE states of **RB‐NI** share similar energy levels, we did not find any ^3^CT→^3^LE process in the global analysis of the transient absorption data. This result may be due to the slow kinetics as a result of the endoergic feature. For **RB‐NI‐N** (Scheme S2), CS (20 ps) occurs upon photoexcitation, then the triplet state of NI moiety is produced by SOCT‐ISC (1 ns), however no further CS in the triplet state was observed, so that the low‐lying state is the ^3^NI* (114.2 μs in deaerated toluene), not a CT state.

## Conclusion

In summary, we proposed a novel spiro electron donor/acceptor compact dyad structural profile to access the long‐lived triplet charge‐transfer state (^3^CT), using a rhodamine unit as electron donor (lactam form; RB) and a naphthalimide (NI) unit as electron acceptor. The long‐lived CT state is based on electron spin control, that is, to initiate the final charge separation (CS) with a triplet precursor, which is achieved with the spin orbit charge transfer intersystem crossing (SOCT‐ISC), without invoking of any heavy‐atom effect or a chromophore with intrinsic ISC ability. The novel spiro linker is rigid and short, thus a well‐defined close‐to‐orthogonal geometry is attained in the dyad (confirmed with single‐crystal X‐ray diffraction molecular structure determination), beneficial for the SOCT‐ISC process. We observed fast ^1^NI*→^1^CT CS process (2.5 ps), and the subsequent charge recombination (CR; that is, SOCT‐ISC) to give the ^3^NI state, that is, ^1^CT→^3^NI* (8 ns). Owing to the small driving force for the secondary CS with ^3^NI* state as the precursor, as well as the weak electronic coupling between the donor and acceptor, we observed a slow secondary CS (125 ns) with ^3^NI as the precursor, that is, ^3^NI*→^3^CT. Control experiments confirmed this is a slow intramolecular CS process, not an intermolecular process. The final CT state is long‐lived (0.94 μs) and exhibits a high energy level (ca. 2.12 eV) in fluid solution at room temperature. These cascade photophysical processes are summarized as ^1^NI*→^1^CT→^3^NI*→^3^CT. Note the electron spin control for accessing long‐lived ^3^CT state is realized. Time‐resolved electron paramagnetic resonance (TREPR) spectra indicated ^3^LE state, with electron spin polarization (ESP) of EEEAAA, via SOCT‐ISC mechanism. Radical pair ISC (RP ISC) is excluded based the ESP pattern. To our knowledge, it is the first report that a long‐lived CT state is generated in a compact electron donor/acceptor dyad via an electron spin‐control strategy implemented by SOCT‐ISC, instead of the previously reported method based on transition metal complexes or chromophores showing intrinsic ISC ability, thus some of the limitations of the conventional methods for accessing long‐lived CT state are addressed. Our results present a new method to design simple, compact electron donor/acceptor dyads to access long‐lived CT states with a high energy level, which is important for artificial photosynthesis, photovoltaics, photocatalysis and as well as for fundamental photochemistry studies.

## Conflict of interest

The authors declare no conflict of interest.

## Supporting information

As a service to our authors and readers, this journal provides supporting information supplied by the authors. Such materials are peer reviewed and may be re‐organized for online delivery, but are not copy‐edited or typeset. Technical support issues arising from supporting information (other than missing files) should be addressed to the authors.

Supplementary
