# Supplementary material for: Long‐Lived Charge‐Transfer State Induced by Spin‐Orbit Charge Transfer Intersystem Crossing (SOCT‐ISC) in a Compact Spiro Electron Donor/Acceptor Dyad
Source: Angew Chem Int Ed Engl. 2020 May 8;59(28):11591–9. doi: 10.1002/anie.202003560 (PMC7496792; doi:10.1002/anie.202003560)
Supplement: Supplementary file 1 — Supplementary [file ANIE-59-11591-s001.pdf]

Angewandte Chemie
Eine Zeitschrift der Gesellschaft Deutscher Chemiker

Supporting Information

Long-Lived Charge-Transfer State Induced by Spin-Orbit Charge Transfer Intersystem Crossing (SOCT-ISC) in a Compact Spiro Electron Donor/Acceptor Dyad

Dongyi Liu, Ahmed M. El-Zohry, Maria Taddei, Clemens Matt, Laura Bussotti, Zhijia Wang, Jianzhang Zhao, Omar F. Mohammed,* Mariangela Di Donato,* and Stefan Weber**

anie_202003560_sm_miscellaneous_information.pdf

Table of Contents

1. Experimental Details……………...……………………………………………………………………...............2

2. Molecular Structure Characterization Data……………………………………………………….……..……6

3. Crystal Data of **RB-NI** and **RB-NI-N.**……………………………………………….............................12

4. DFT Calculations……………..…………………..……………………………………………………..…..…..14

5. Fluorescence Emission Spectra………………………………………………………………..................16

6. Singlet Oxygen Quantum Yield………………………………………………………………………..…17

7. Cyclic Voltammogram of the Compounds and Spectroelectrochemistry………………………………18

8. Femtosecond Time-resolved Transient Absorption Spectra………………………………….…………21

9. Nanosecond Time-resolved Transient Absorption Spectra………………..…….…….…………………24

10. Time-resolved Electron Paramagnetic Resonance Data……………………….………………………31

11. Excited State Energy Diagram of **RB-NI-N** ……………………………………………………………..…31

References……………...………………………..……………………………………………….................32

1. Experimental Details

General Methods. UV–vis absorption spectra were measured on an Agilent 8453 UV-vis spectrophotometer. Fluorescence emission spectra were recorded on a Shimadzu RF-5301PC spectrofluorometer. Luminescence lifetimes were measured on an OB920 fluorescence/ phosphorescence lifetime spectrometer (Edinburgh Instruments, U.K.). The synthesis of compounds **1**–**4** and **NI-NH$_2$** were based on the reported methods,[1] all the chemicals used in the synthesis are analytically pure. ^{1}H NMR and ^{13}C NMR spectra were recorded on the Bruker Avance spectrometers (400 MHz). ^{1}H and ^{13}C chemical shifts were reported in parts per million (ppm) relative to TMS, with the residual solvent peak used as an internal reference. The mass spectra were measured by HRMS (MALDI-TOF).

Femtosecond Time-Resolved Transient Absorption Spectroscopy. The setup used for the fs transient absorption spectroscopy (TAS) measurements is based on a Ti:sapphire regenerative amplifier (BMI Alpha 1000) system pumped by a Ti:sapphire oscillator (Spectra Physics Tsunami). The system produces 100 fs pulses at 785 nm, 1 kHz repetition rate and average power of 450–500 mW. Excitation pulses at 400 nm have been produced as second harmonic of the fundamental laser beam by using a BBO crystal. The pump beam polarization has been set to magic angle with respect to the probe beam by rotating a λ/2 plate. Excitation powers were on the order of 50-80 nJ. The probe pulse was generated by focusing a small portion of the fundamental laser output radiation on a 2 mm thick calcium fluoride windows, kept under continuous rotation through a motorized holder. Before light generation, the portion of fundamental beam used for the probe beam was delayed with respect to the pump by motorized stage. After crossing the pump beam at the sample position, the white light continuum was sent to a flat field monochromator coupled to a home-made CCD detector. For all measurements the sample was placed in a quartz cell (2 mm thick) mounted on a movable stage to avoid sample photo degradation and multiple photon excitation. The recorded kinetic traces and transient spectra have been analysed by using a global analysis.[2,3] The number of kinetic components has been estimated by performing a preliminary singular values decomposition (SVD) analysis. [2] Global analysis was performed using the GLOTARAN package (http://glotaran. org),[3] and employing a linear unidirectional "sequential" model.

Sub-nanosecond Time-Resolved Transient Absorption Spectroscopy. The sub-ns TA experiment was carried out using an ultrafast Systems EOS UV-NIR spectrometer with broadband capability and time resolutions of 800 ps. In EOS experiment, a white-light continuum probe pulse that was generated by a super continuum source, and spectrally tunable (240−2600 nm) ultrafast pump pulses were generated in an optical parametric amplifier (Newport Spectra-Physics). The pump and probe beams were focused on the sample solution, and the transmitted probe light from the sample was collected and focused on the broadband UV−visible detector to record the time-resolved transient absorption spectra. More details see elsewhere.[4]

Nanosecond Time-Resolved Transient Absorption Spectroscopy. LP980 laser lash photolysis spectrometer (Edinburgh Instruments Ltd., UK) was used to record the ns TA spectra of the compounds. Optical parametric oscillator (OPO, tunable between 210–710 nm) was used as the pulse laser excitation source, and xenon probe source was a 150 W ozone-free lamp, Xe arc lamp (pulsed mode up to 10 Hz). The sample was excited by the OPO pump laser beam and probe beam from a xenon lamp was passed through the sample at right angles to the path of the exciting pulse (cross beam configuration). After passing through the sample the probe beam was directed to a monochromator. The transmission properties of the sample before, during, and after the exciting pulse were converted by the detector into electrical signals that were measured by an oscilloscope (TDS 3012C, 100 MHz). For all measurements the sample was placed in a quartz cuvette (10 mm thick) mounted on an immovable stage. Before measurements, all sample solutions were bubbled with N_2 for ca. 15 min. To measure the transient absorption of $NI^{-\bullet}$, the samples (without TEA) were deaerated with N_2 for ca. 15 min, then TEA was added and bubbled with N_2 for ca. 5 min before measurement. The N_2 flow was maintained during the deaerated ns TA measurements. The recorded kinetic traces and transient spectra have been analysed by using a LP900 software.

Time-Resolved Electron Paramagnetic Resonance (TREPR) Spectroscopy. X-band TREPR spectra were recorded following pulsed laser excitation (355 nm and 427 nm) of the respective sample performed using an Ekspla NT342 (EKSPLA, Vilnius, Lithuana) integrated Nd:YAG/OPO laser system that produces laser pulses of 3–5 ns pulse duration at a repetition rate of 20 Hz. A laboratory-build EPR spectrometer was used in combination with a Bruker microwave bridge ER046 MRT (Bruker, Billerica, MA). The sample was placed in a synthetic quartz (Suprasil) tube with an inner diameter of about 3 mm, and irradiated in a dielectric-ring resonator ER 4118x-MD5 (Bruker). The resonator was immersed into a helium gas-flow cryostat and EPR data collected at temperatures adjusted and stabilized by an Oxford ITC503 temperature controller (Oxford, United Kingdom). The microwave power was set to 2 mW and the microwave frequency controlled by a frequency counter Keysight 5352B (Keysight Technologies, Böblingen, Germany). Signal acquisition was performed with a transient recorder LeCroy 9354A (Chestnut Ridge, NY) at a detection bandwidth of 25 MHz. Distortions of the signal baseline introduced by the laser pulse were corrected by subtracting a signal collected at an off-resonant magnet field position.

X-Ray Diffraction. X-ray diffraction data of **RB-NI** and **RB-NI-N** were collected on a Bruker SMART APEX CCD diffractometer with graphite-monochromated M*o* Kα (λ = 0.71073 Å) at 296 K, using the SMART and SAINT programs. The crystal structure refinement and analysis were performed on the Olex2 1.2 program.[5] The crystal data and structure refinement for **RB-NI** and **RB-NI-N** are presented in the Supporting Information (Table S1 and S2). CCDC_1956134 (**RB-NI**) and CCDC 1956097 (**RB-NI-N**) contain the supplementary crystallographic data for this paper and the data can be obtained freely from the Cambridge Crystallographic Data Centre via https://www.ccdc.cam.ac.uk/.

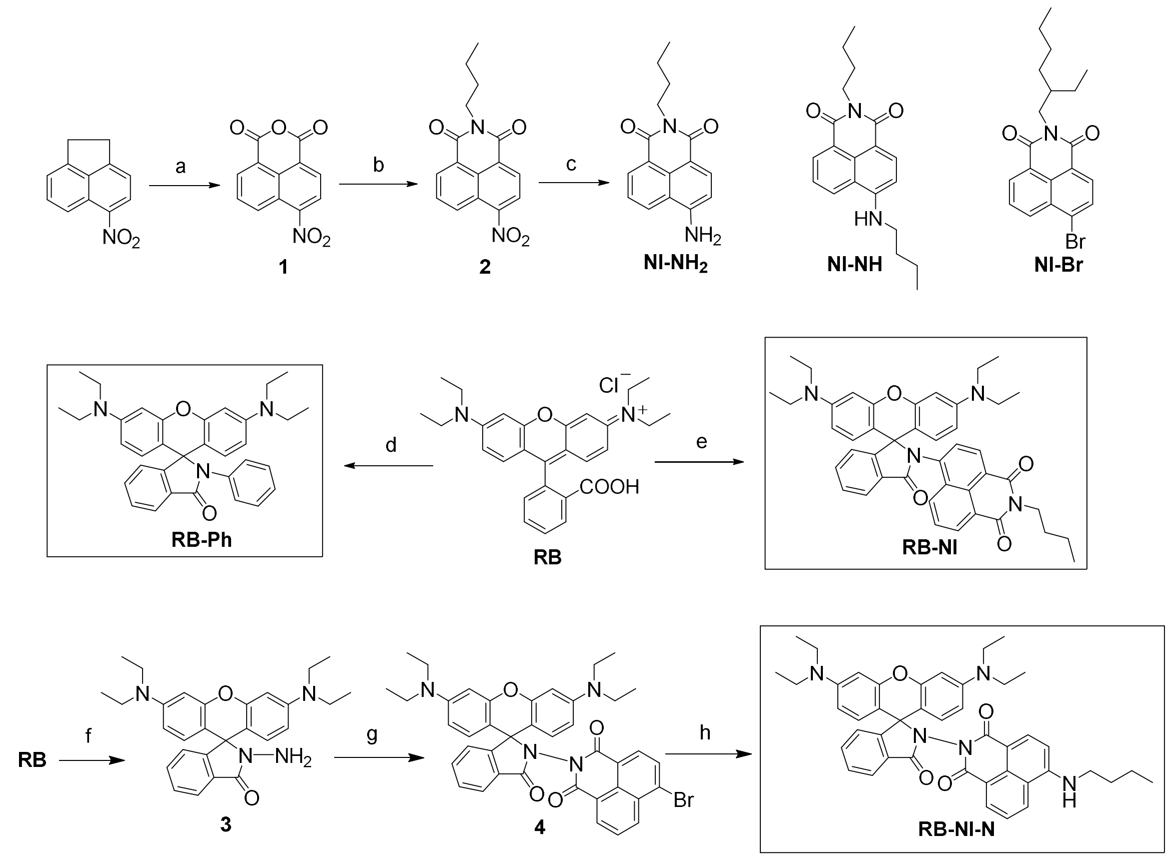

Scheme S1. Synthesis of RB Derivatives. Keys: (a) $Na_2CrO_7{\cdot}2H_2O$, AcOH, reflux, 5 h, yield: 79%. (b) *n*-butyl amine, EtOH, reflux, 2 h, yield: 96%. (c) $SnCl_2{\cdot}2H_2O$ in 12 mol/L HCl, EtOH, rt, 20 min, 10% Na_2CO_3, yield: 58%. (d) $POCl_3$, dry DCM, reflux, 5 h; aniline (excess), CH_3CN, Et_3N, reflux, 9 h; yield: 46%. (e) $POCl_3$, dry DCM, reflux, 5 h; **NI-NH₂**, CH_3CN, Et_3N, reflux, 21 h; yield: 43%. (f) $NH_2NH_2{\cdot}H_2O$, EtOH, reflux, 4.5 h, yield: 87%. (g) 6-bromo-1*H*,3*H*-benzo[de]isochromene-1,3-dione, AcOH, reflux, 24 h, yield: 77%. (h) *n*-butyl amine, reflux, 20 h, yield: 39%.

Synthesis of Compound RB-NI. Rhodamine B (80 mg, 0.18 mmol) was dissolved in dry DCM (5 mL), then $POCl_3$ (0.1 mL) was added under N_2 atmosphere. The mixture was refluxed and stirred for 5 h under N_2. The solvent was removed under reduced pressure, then crude product was dissolved in dry CH_3CN (8 mL). NI-NH_2 (27 mg, 0.1 mmol) and Et_3N (0.2 mL) was added under N_2, then the mixture was refluxed and stirred for 21 h under N_2. After the reaction was done, the solvent was removed under reduced pressure and the crude product was purified by column chromatography (silica gel, DCM : ethyl acetate = 25:1, v/v). Compound RB-NI was obtained as canary yellow solid. Yield: 30 mg (43%). M. p.: 198.3–200.0 °C; ^{1}H NMR (400 MHz, $CDCl_3$, ppm) δ: 8.42 (d, J = 4.0 Hz, 1H), 8.29 (d, J = 8.0 Hz, 1H), 8.10 (d, J = 4.0 Hz, 1H), 7.63–7.73 (m, 3H), 7.37–7.41 (m, 2H), 6.70–6.74 (m, 2H), 6.57 (d, J = 8.0 Hz, 1H), 6.45 (d, J = 8.0 Hz, 1H), 6.22 (s, 2H), 5.87 (s, 1H), 4.12 (t, J = 8.0 Hz, 2H), 3.38 (q, J = 8.0 Hz, 4H), 3.16 (q, J = 8.0 Hz, 4H), 1.64–1.71 (m, 2H), 1.38–1.47 (m, 2H), 1.20 (t, J = 8.0 Hz, 6H), 0.94–1.01 (m, 9H); ^{13}C NMR ($CDCl_3$, 100 MHz): δ = 167.0, 164.2, 163.9, 154.4, 153.7, 151.2, 149.3, 148.9, 139.5, 133.2, 132.2, 131.0, 130.9, 130.7, 129.8, 129.0, 128.9, 128.8,

128.7,127.6, 126.1, 124.7, 123.8, 122.6, 122.2, 108.4, 107.7,106.7, 106.0, 97.9, 97.6, 77.3, 77.0, 76.7, 69.6, 44.5, 44.3, 40.2, 30.2, 20.4, 13.8, 12.5, 12.3. MALDI–HRMS ($C_{44}H_{44}N_4O_4 + H^+$): Calcd, $m/z = 693.3441$; found, $m/z = 693.3438$.

Synthesis of Compound RB-NI-N. Compound **4** (72 mg, 0.11 mmol) was dissolved in *n*-butylamine (10 mL), then the mixture was refluxed and stirred for 20 h under N_2. After the reaction was completed, the solvent was removed under reduced pressure and the crude product was purified by column chromatography (silica gel, DCM : ethyl acetate, from 1:0 to 6:1 gradually, v/v). Compound **RB-NI-N** was obtained as yellow solid. Yield: 30 mg (39%). M. p.: >250.0°C; ^{1}H NMR (400 MHz, $CDCl_3$, ppm) δ: 8.16 (d, J = 8.0 Hz, 1H), 8.07 (d, J = 8.0 Hz, 1H), 7.72–7.75 (m, 1H), 7.60–7.68 (m, 2H), 7.55–7.58 (m, 1H), 7.33 (d, J = 8.0 Hz, 1H), 7.00–7.02 (m, 1H), 6.68–6.72 (m, 2H), 6.32–6.37 (m, 3H), 6.21–6.22 (m, 1H), 6.08 (d, J = 4.0 Hz, 1H), 6.04 (d, J = 4.0 Hz, 1H), 3.17–3.33 (m, 10H), 1.68–1.74 (m, 2H), 1.44–1.52 (m, 2H), 1.03–0.09 (m,12H), 0.97 (t, J = 12.0 Hz, 3H); ^{13}C NMR ($CDCl_3$, 100 MHz): δ = 161.3, 160.6, 154.5, 150.7, 150.4, 148.8, 134.1, 133.1, 131.1, 131.0, 130.8, 130.3, 128.8, 128.5, 127.2, 124.8, 124.0, 123.2, 121.4,119.9, 108.0, 107.6, 105.9, 105.6, 103.2, 97.6, 77.4, 77.2, 77.0, 76.7, 68.2, 53.4, 44.3, 43.5, 30.4, 20.4, 14.0, 12.5. MALDI–HRMS ($C_{44}H_{45}N_5O_4 + H^+$): Calcd, $m/z = 708.3550$; found, $m/z = 708.3558$.

Synthesis of Compound RB-Ph. Rhodamine B (75 mg, 0.17 mmol) was dissolved in dry DCM (10 mL), then $POCl_3$ (0.1 mL) was added under N_2 atmosphere. The mixture was refluxed and stirred for 5 h under N_2. The solvent was removed under reduced pressure, then crude product was dissolved in dry CH_3CN (8 mL). Aniline (0.1 mL, 1 mmol) was added under N_2 and the mixture was stirred for 15 min at room temperature, then the mixture was refluxed and stirred for 9 h under N_2. After the reaction was completed, the solvent was removed under reduced pressure and the crude product was purified by column chromatography (silica gel, DCM : ethyl acetate = 20:1, v/v). **RB-Ph** was obtained as white solid. Yield: 40 mg (46%). M.p.: 227.5–229.5°C; ^{1}H NMR (400 MHz, $CDCl_3$, ppm) δ: 8.0 (s, 1H), 7.49 (s, 2H), 7.10–7.14 (m, 4H), 6.80 (d, J = 4.0 Hz, 2H), 6.64 (d, J = 8.0 Hz, 2H), 6.30 (d, J = 8.0 Hz, 2H), 6.25 (s, 2H), 3.30–3.32 (m, 8H), 1.14 (m, 12H); ^{13}C NMR ($CDCl_3$, 100 MHz): δ = 167.7, 153.3, 153.2, 148.8, 136.7, 132.8, 131.1, 128.9, 128.5, 128.1, 127.3, 126.6, 124.0, 123.4, 108.2, 106.5, 97.9, 77.4, 77.0,76.7, 67.5, 44.3, 12.6; ESI–HRMS ($C_{34}H_{35}N_3O_2 + H]^+$): Calcd, $m/z = 518.2808$; found, $m/z = 518.2819$.

2. Molecular Structure Characterization Data

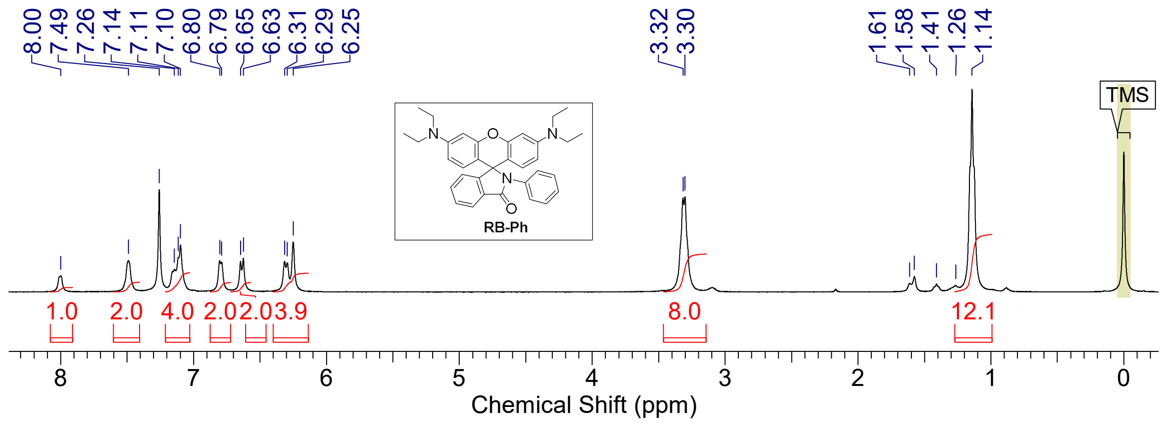

Figure S1. ^{1}H NMR spectrum of compound **RB-Ph** ($CDCl_3$, 400 MHz).

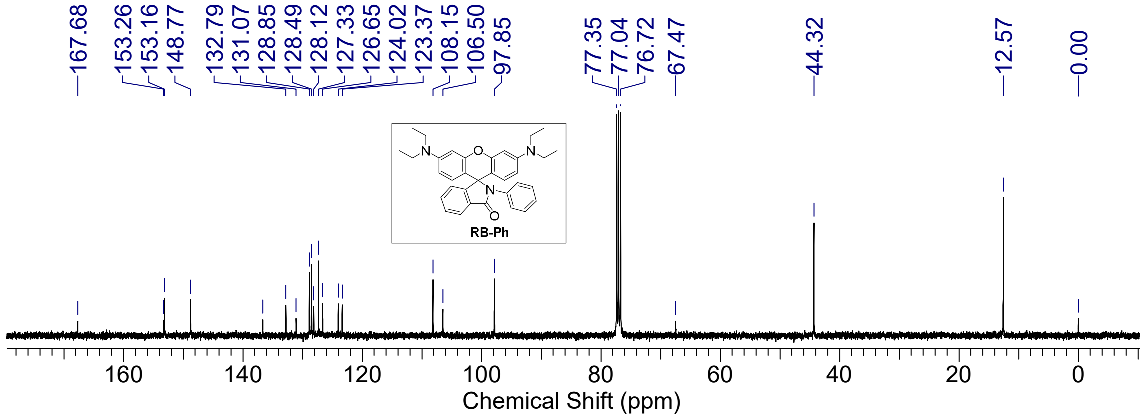

Figure S2. ^{13}C NMR spectrum of compound **RB-Ph** ($CDCl_3$, 126 MHz).

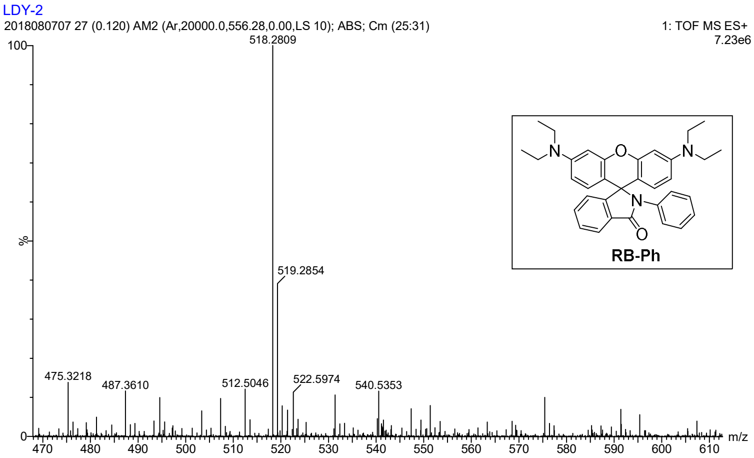

Elemental Composition Report

Single Mass Analysis

Tolerance = 5.0 PPM / DBE: min = -1.5, max = 100.0

Element prediction: Off

Number of isotope peaks used for i-FIT = 9

Monoisotopic Mass, Odd and Even Electron Ions

4 formula(e) evaluated with 1 results within limits (up to 50 closest results for each mass)

Elements Used:

C: 1-50 H: 1-50 N: 3-3 O: 2-2

Minimum:				-1.5				
Maximum:		5.0	5.0	100.0				
Mass	Calc. Mass	mDa	PPM	DBE	i-FIT	Norm	Conf(%)	Formula
518.2809	518.2808	0.1	0.2	18.5	1411.7	n/a	n/a	C34 H36 N3 O2

Figure S3. TOF-HRMS spectrum of compound **RB-Ph**.

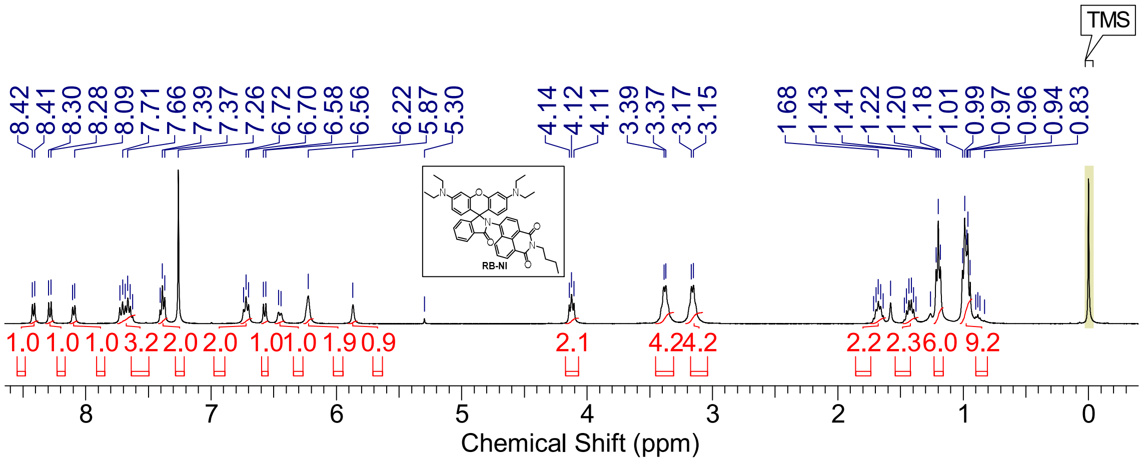

Figure S4. ^{1}H NMR spectrum of compound **RB-NI** ($CDCl_3$, 400 MHz).

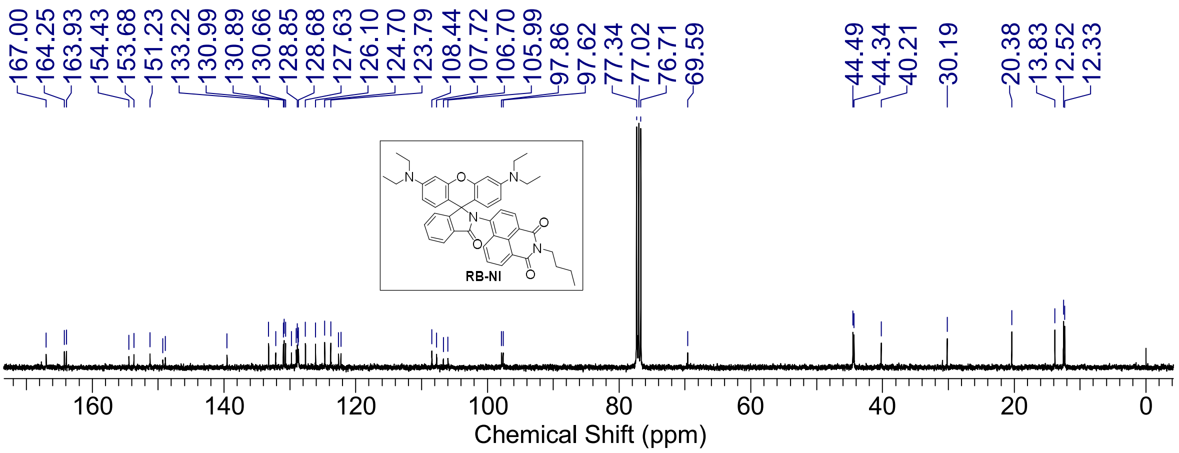

Figure S5. ^{13}C NMR spectrum of compound **RB-NI** ($CDCl_3$, 126 MHz)

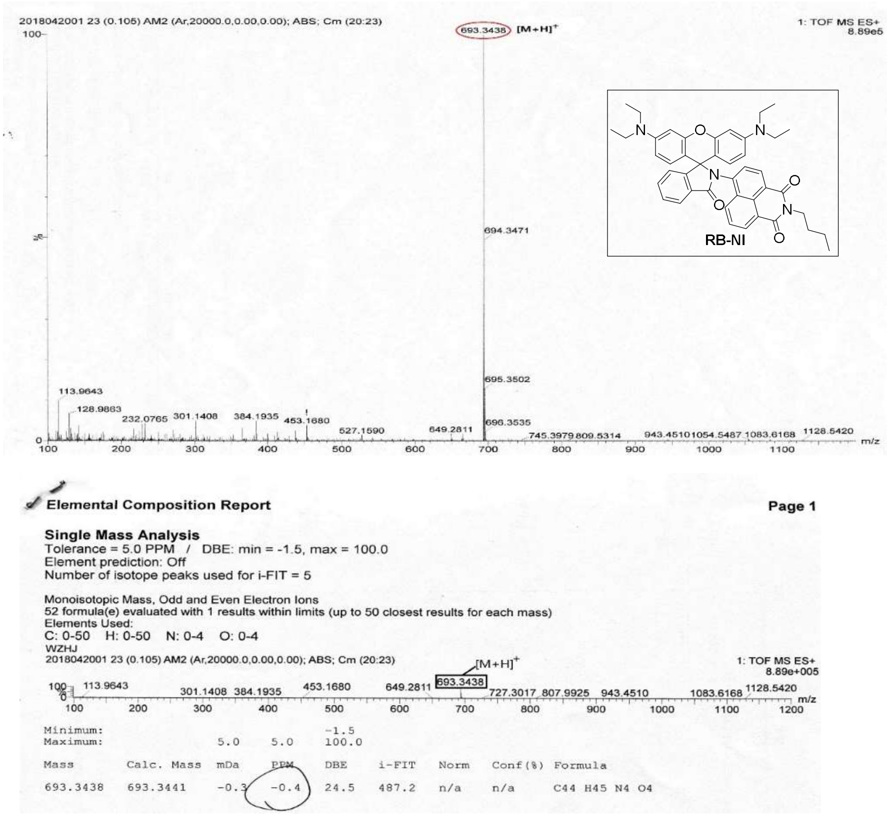

Figure S6. TOF-HRMS spectrum of compound **RB-NI.**

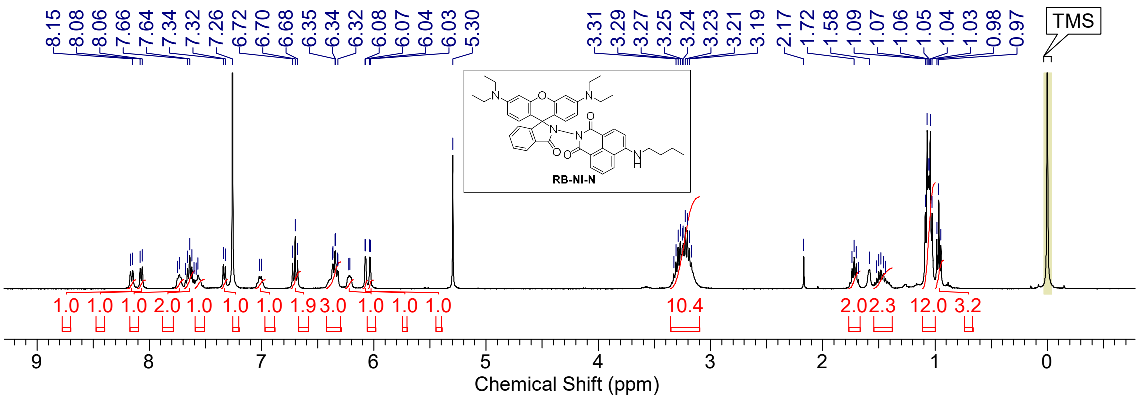

Figure S7. ^{1}H NMR spectrum of compound **RB-NI-N**. ($CDCl_3$, 400 MHz).

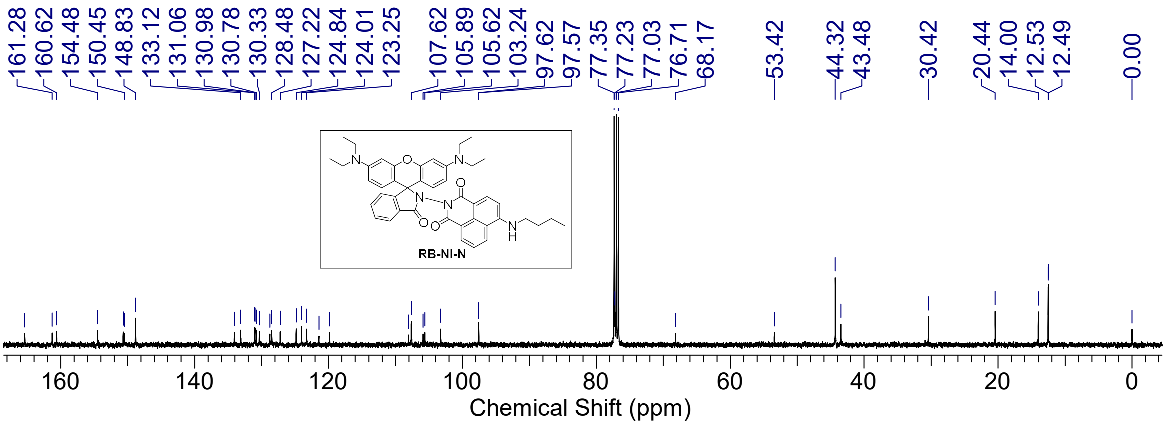

Figure S8. ^{13}C NMR spectrum of compound **RB-NI-N** ($CDCl_3$, 126 MHz).

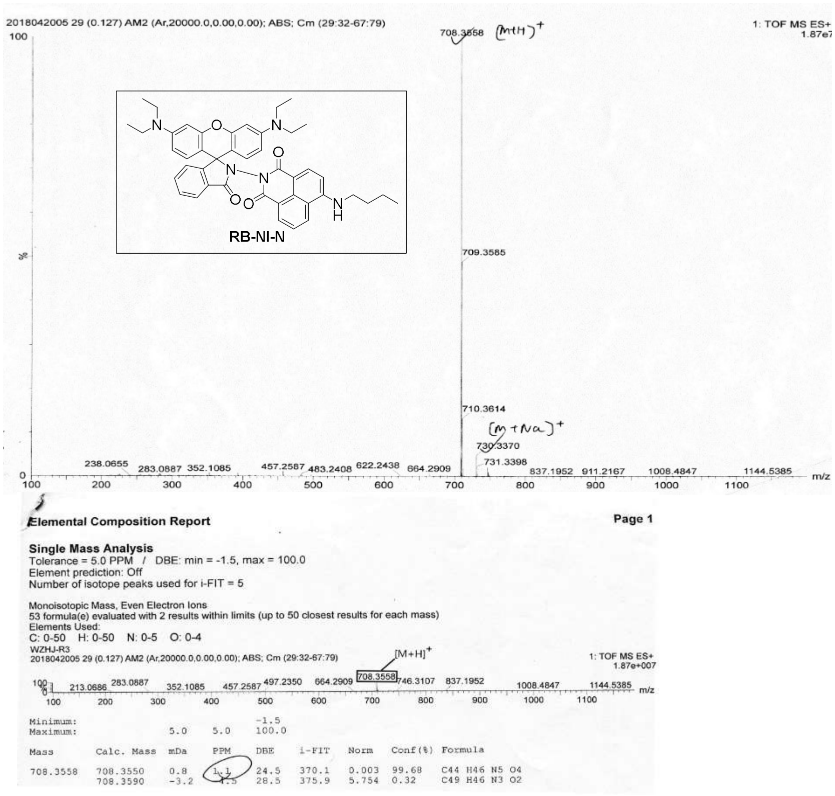

Figure S9. TOF-HRMS spectrum of compound **RB-NI-N.**

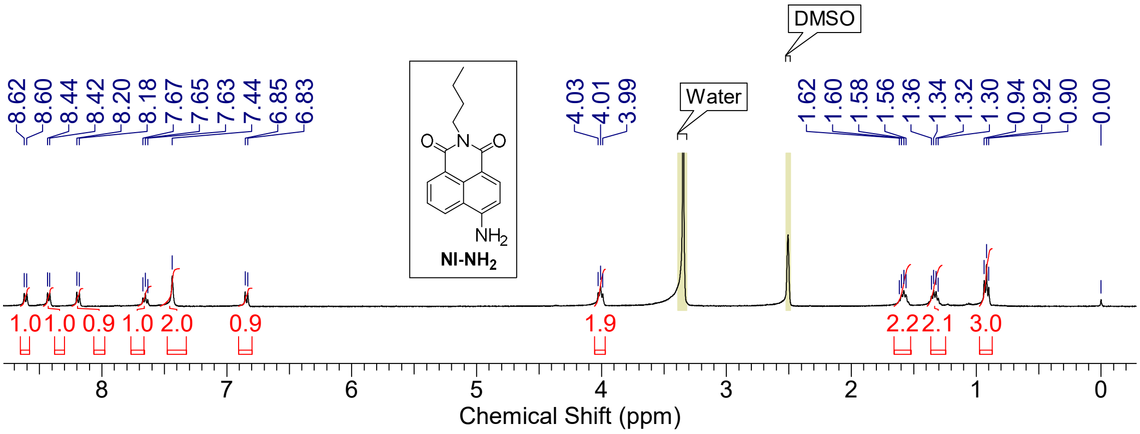

Figure S10. ^{1}H NMR spectrum of Compound **NI-NH$_2$** (DMSO-d_6, 400 MHz).

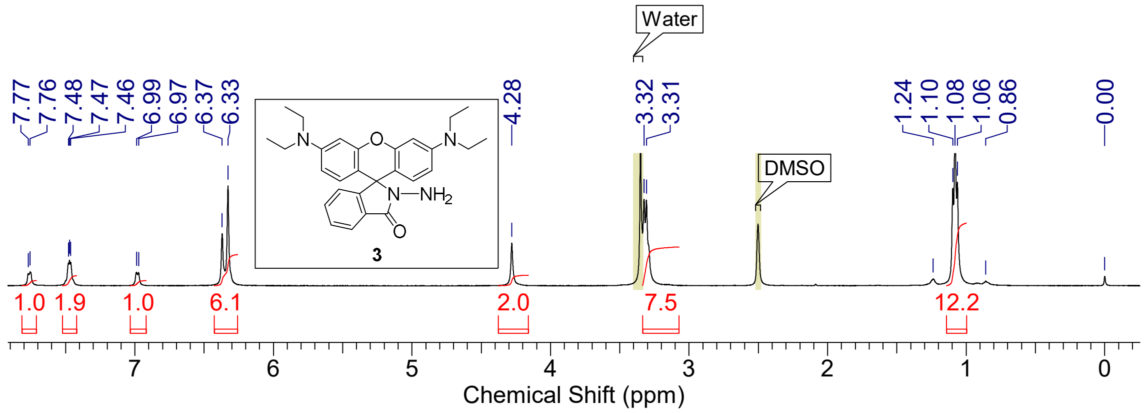

Figure S11. ^{1}H NMR spectrum of Compound **3** (DMSO-d_6, 400 MHz).

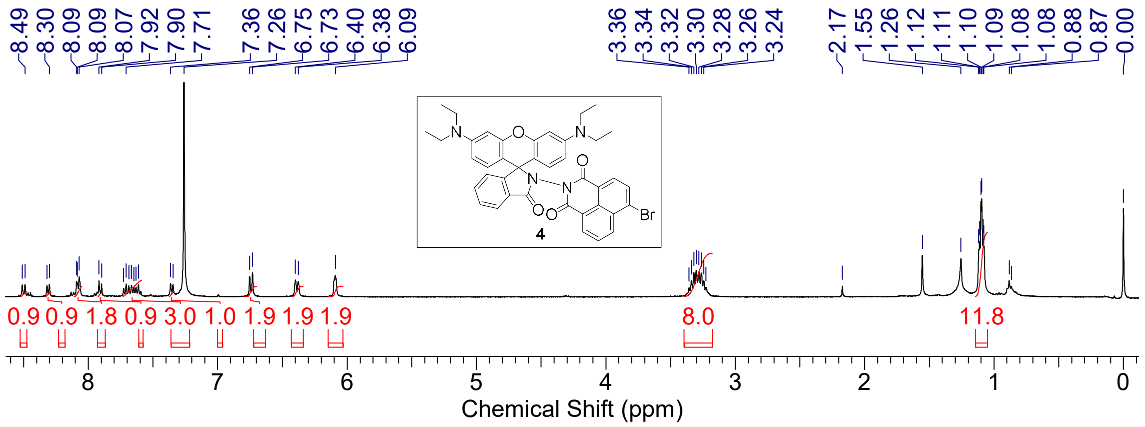

Figure S12. ^{1}H NMR spectrum of Compound **4** ($CDCl_3$, 400 MHz).

3. Crystal Data of RB-NI and RB-NI-N

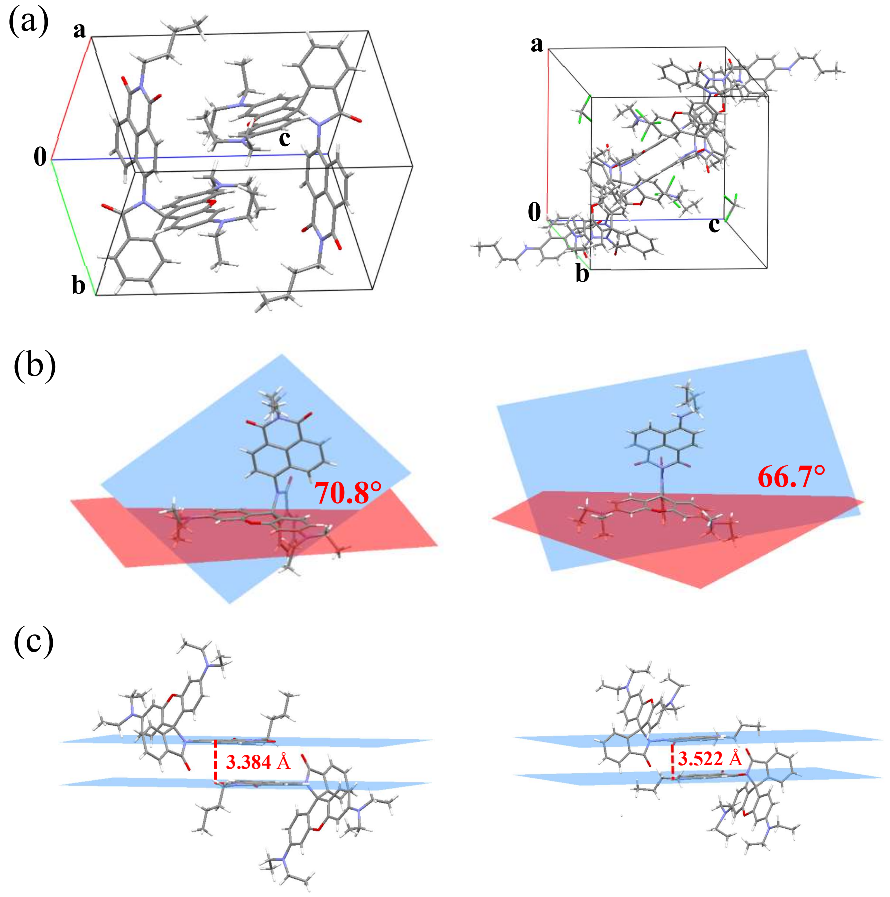

Figure S13. (a) The unit cell structure of the crystal **RB-NI** (left) and **RB-NI-N** (right). (b) Dihedral angle between xanthene and the NI planes of **RB-NI** (left) and **RB-NI-N** (right), the two color sheets show the planes of the NI and the RB moieties, respectively. (c) The vertical distances between the adjacent NI planes in the single crystals of **RB-NI** (left) and **RB-NI-N** (right), blue sheets show the plane of adjacent NI moieties

We observed weak π–π intermolecular interactions between the adjacent NI moiety in the single crystals, the vertical distance between the planes are 3.384 Å and 3.522 Å in **RB-NI** and **RB-NI-N**, respectively (Figure S13c).

Table S1. Crystal Data and Structure Refinement for **RB-NI**.

Moiety formula	$C_{44}H_{44}N_4O_4$
Sum formula	$C_{44}H_{44}N_4O_4$
Sum formula weight	692.83
Crystal system	Triclinic
Space group	$P\bar{1}$
a (Å)	9.940(4)
b (Å)	12.250(4)
c (Å)	15.711(7)
α (deg)	78.43(3)
β (deg)	75.39(3)
γ (deg)	83.36(3)
Temperature / K	296
Volume / Å^3	1809.5(12)
Z	2
2θ range for data collection (deg)	3.96–49.996
Mμ (mm^{-1})	0.082
D_x (g/cm^3)	1.272
F_{000}	736.0
Crystal size/mm^3	0.15×0.13×0.1
h, k, l_{max}	11, 14, 18
Reflections collected	11019
Independent reflections	6183
Data/restraints/parameters	6183/1147/475
T_{min}, T_{max}	0.524, 0.746
Largest diff. peak/hole (e Å^3)	0.783/–0.459
Data completeness	0.970
R_1, wR_2 [I ≥ 2σ(I)]	0.1020, 0.2898
R_1, wR_2 (all data)	0.1858, 0.3429
Goodness of fit	1.007
CCDC number	1956134

Table S2. Crystal Data and Structure Refinement for **RB-NI-N**.

Moiety formula	$CHCl_3$, $C_{44}H_{45}N_5O_4$
Sum formula	$C_{45}H_{46}N_5O_4$
Sum formula weight	827.22
Crystal system	Monoclinic
Space group	$P2_1/n$
a (Å)	16.401(8)
b (Å)	15.675(8)
c (Å)	16.674(8)
α (deg)	90
β (deg)	93.480(9)
γ (deg)	90
Temperature / K	296
Volume / Å^3	4279(4)
Z	4
2θ range for data collection (deg)	3.594–49.998
Mμ (mm^{-1})	0.263
D_x (g/cm^3)	1.284
F_{000}	1736.0
Crystal size/mm^3	1738.40
h, k, l_{max}	19, 18, 19
Reflections collected	23708
Independent reflections	7513
Data/restraints/parameters	7513/1/520
T_{min}, T_{max}	0.581, 0.746
Largest diff. peak/hole (e Å^3)	0.494/–0.613
Data completeness	0.998
R_1, wR_2 [I ≥ 2σ(I)]	0.0900, 0.2435
R_1, wR_2 (all data)	0.2090, 0.2947
Goodness of fit	0.962
CCDC number	1956097

4. FT Calculations

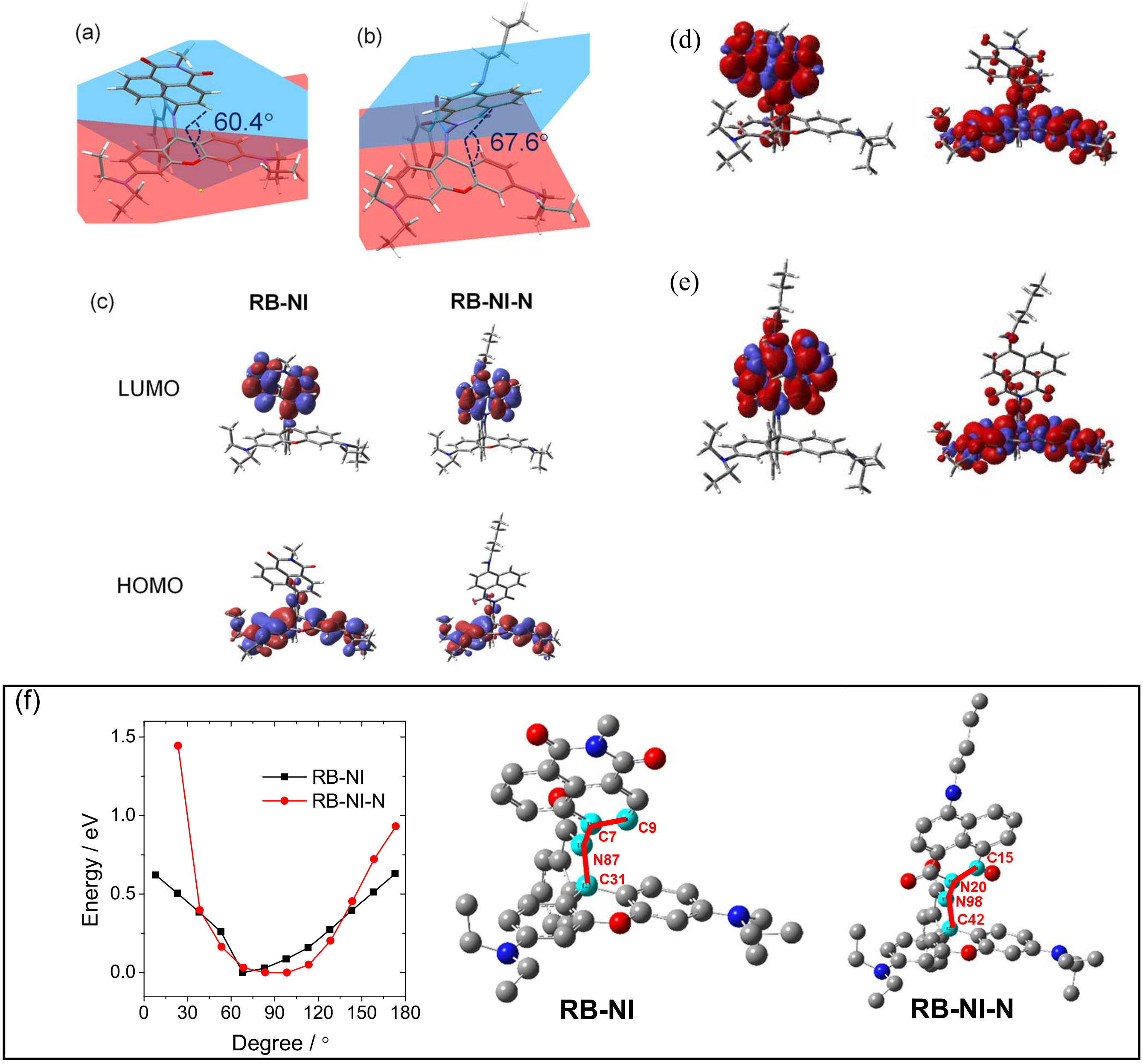

Figure S14. Optimized ground state conformations of (a) **RB-NI** and (b) **RB-NI-N**; the two color sheets show the planes of the NI and the RB moieties, respectively. (c) The highest occupied molecular orbital (HOMO) and lowest unoccupied molecular orbital (LUMO) of **RB-NI** and **RB-NI-N**. Spin density distributions of optimized radical ground state of (d) **RB-NI** (left picture: charge = –1, right picture: charge = 1), (e) **RB-NI-N** (left picture: charge = –1, right picture: charge = 1). (f) Ground state potential energy curves of **RB-NI** and **RB-NI-N**, as a function of the rotational dihedral angle of the selected atoms. All the calculations were performed at the DFT (B3LYP/6−31G(d)) level with Gaussian 09W.

All the calculations (geometry optimizations, molecule orbitals and spin density distributions) of the compounds were performed at the density functional theory (DFT) based on (B3LYP/6−31G(d)). The Gaussian 09 program package was used for the calculations.[6] The spin density distributions of optimized radical anion and radical cation ground states of **RB-NI** and **RB-NI-N** (Figure S28) demonstrates that the radical anion and cation are localized on the NI and RB moieties, respectively. The ground state potential energy curves (PECs) of RB-NI and RB-NI-N are shown in Figure S14. The PEC generated by rotation about the C−N bond of the RB-NI shows significant steric hindrance, however, the PEC generated by rotation about the N−N bond in RB-NI-N shows less conformational restriction.

Table S3. Main Transition Orbitals, Transition Wavelengths *λ*calc and Oscillator Strengths *f* of Singlet Excited States. Calculation Was Performed at the B3LYP/6-31G(d) Level with Gaussian 09.

Compound	state	Energy	Composition	*f*	Character
**RB-Ph**	S1	3.76 eV / 330 nm	H→L	0.0051	CT
	S2	3.79 eV / 327nm	H–1→L	0.0026	CT
	S3	4.28 eV / 290 nm	H→L+1	0.0043	LE
	T1	3.38 eV / 367 nm	H–2→L	0.0000	LE
	T2	3.41 eV / 363 nm	H→L+2	0.0000	LE
	T3	3.50 eV / 355 nm	H–1→L+2	0.0000	LE
**RB-NI**	S1	2.67 eV / 465 nm	H→L	0.0067	CT
	S2	2.82 eV / 440 nm	H–1→L	0.0051	CT
	S3	3.31 eV / 375 nm	H–2→L	0.0060	CT
	T1	2.25 eV / 552 nm	H–3→L	0.0000	CT&LE
	T2	2.67 eV / 464 nm	H→L	0.0000	CT
	T3	2.80 eV / 442 nm	H–1→L	0.0000	CT
**RB-NI-N**	S1	2.51 eV / 495nm	H→L	0.0061	CT
	S2	2.58 eV / 480nm	H–1→L	0.0001	CT
	S3	3.06 eV / 405nm	H–2→L	0.0003	CT
	T1	2.21 eV / 562 nm	H–3→L	0.0000	LE
	T2	2.50 eV / 495 nm	H→L	0.0000	CT
	T3	2.58 eV / 481 nm	H–1→L	0.0000	CT

5. Fluorescence Emission Spectra

In Figure S15, the emission lifetime shows mono-exponential decay and do not prolong (~15 ns) when raising temperature, which are differ from thermaly activated delayed fluorescence (TADF. Usually show double-exponential decay of the emission and with a long lifetime component). Hence, TADF is excluded for **RB-NI**.

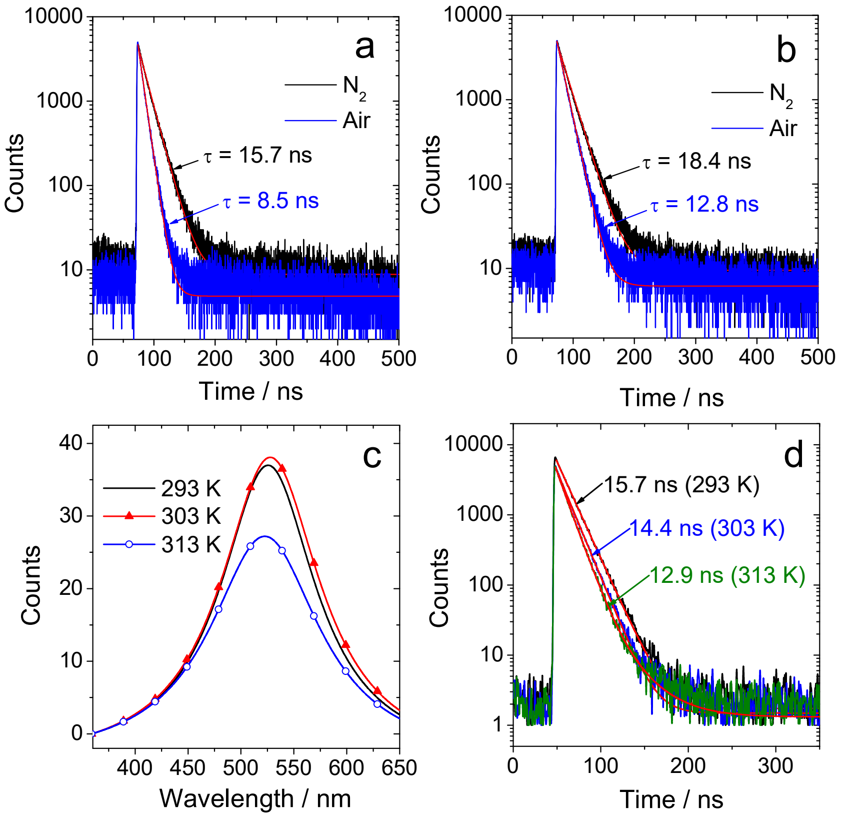

Figure S15. Luminescence lifetime spectra were compared in N_2 and air saturated solutions: (a) **RB-NI** (*c* = 2.0 × 10^{-5} M) and (b) **RB-NI-N** (*c* = 5.0 × 10^{-5} M), in *n*-hexane, λ_{ex} = 340 nm, 20 °C. Luminescence emission spectra of **RB-NI** vary from 293 K to 313 K: (c) emission and (d) lifetimes obtained at 540 nm, *c* = 2.0 × 10^{-5} M in *n*-hexane, λ_{ex} = 340 nm.

6. Singlet Oxygen Quantum Yield

Table S4. Singlet Oxygen Quantum Yields of RB Derivatives in Different Solvents. [a]

Compounds	*n*-Hexane	Toluene	DCM	MeCN
**RB-Ph** (λ_{ex} = 330 nm)	[b]	[b]	[b]	[b]
**RB-NI** (λ_{ex} = 350 nm)	29.8%	26.4%	[b]	[b]
**RB-NI-N** (λ_{ex} = 400 nm)	24.5%	23.2%	[b]	[b]

[a] $Ru(bpy)_3[PF_6]_2$ was used as standard compound (Φ_Δ = 57% in ACN).[7] [b] Not observed.

Singlet oxygen quantum yield (Φ_Δ) were calculated according to the equation (1):

$$\Phi_{\Delta sam} = \Phi_{\Delta sta}\left(\frac{m_{sam}}{m_{sta}}\right)\left(\frac{F_{sta}}{F_{sam}}\right) \quad (1)$$

where “sam” and “sta” designate the “samples” and “$Ru(bpy)_3[PF_6]_2$”, respectively. “*m*” is the slope of difference in change in absorbance of DPBF (at 410 nm) with the irradiation time, “*F*” is the absorption correction factor, which is given by $F = 1-10^{-OD}$ (OD is the absorbance at the irradiation wavelength). The singlet oxygen quantum yield of reference compounds **NI-NH₂** and **NI-NH** are zero and it means they cannot exhibit efficient ISC ability, which is differ from the NI derivatives (without amino at the 4 position) always show efficient ISC. Therefore, it gives us an implication that the ISC ability of **RB-NI** and **RB-NI-N** is not induced by intrinsic ISC ability of NI moiety, another mechanism plays a vital role in the occurrence of ISC.

7. Cyclic Voltammogram Study of the Compounds and Spectroeletrochemistry

The cyclic voltammogram of compounds were recorded, using a CHI610D electrochemical workstation (Shanghai, China). The electrolytic cell including three electrodes, the glassy carbon electrode as working electrode, the platinum electrode as counter electrode, and the Ag/$AgNO_3$ (0.1 M in acetonitrile) electrode as reference electrode. The electrochemical measurements were performed at room temperature, using $Bu_4N[PF_6]$ (*ca.* 0.1 M) as supporting electrolyte in deaerated DCM, ferrocene as the internal references. The scan rate was 0.1 V/s.

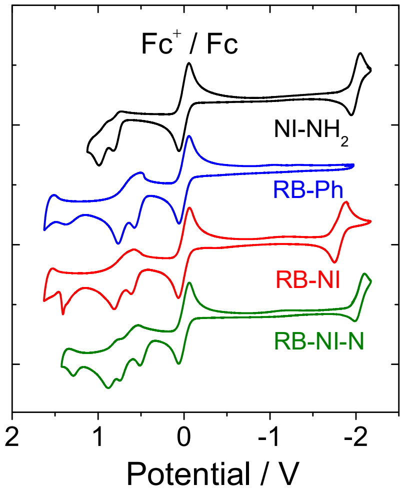

Figure S16. Cyclic voltammograms of the compounds. Ferrocene (Fc) was used as internal reference. Conditions: in deaerated DCM containing $Bu_4N[PF_6]$ (*ca.* 0.1 M) as supporting electrolyte, the redox potentials are versus Fc/Fc^+. Scan rates: 100 mV/s. $c = 1.0 \times 10^{-3}$ M at 20 °C.

Table S5. Electrochemical Parameters, Gibbs Free Energy Changes of the Charge Separation (ΔG_{CS}) and Charge Transfer State Energy Levels (E_{CT}) of the Compounds.[a]

Compounds	$E_{1/2}$(Ox) (V)	$E_{1/2}$(Red) (V)	ΔG_{CS} (eV)/E_{CT} (eV)			
			n-hexane	Toluene	DCM	MeCN
**RB-Ph**	0.54	[b]	[b]	[b]	[b]	[b]
**RB-NI**	0.60	−1.82	−0.85/2.24	−0.88/2.21	−0.97/2.13	−1.00/2.10
**RB-NI-N**	0.52	−2.05	−0.06/2.47	−0.11/2.42	−0.24/2.29	−0.27/2.26
**NI-NH2**	0.78	−2.00	[c]	[c]	[c]	[c]

[a] Cyclic voltammetry in N_2-saturated DCM containing a 0.10 M Bu_4NPF_6, Pt electrode as the counter electrode, glassy carbon electrode as the work electrode, the redox potentials are versus Fc/Fc^+. [b] Not observed. [c] Not applicable.

The Gibbs free energy change (ΔG_{CS}) of the intramolecular electron transfer process and the static Coulombic energy (ΔG_S) can be calculated with the Rehm−Weller equation (2–3). The energy level of charge-transfer states (E_{CT}) were calculated with equation (4).[8]

$$\Delta G_{CS} = e[E_{OX} - E_{RED}] - E_{00} + \Delta G_S \quad (2)$$

$$\Delta G_S = -\frac{e^2}{4\pi\varepsilon_S\varepsilon_0 R_{CC}} - \frac{e^2}{8\pi\varepsilon_0}\left(\frac{1}{R_D} + \frac{1}{R_A}\right)\left(\frac{1}{\varepsilon_{REF}} - \frac{1}{\varepsilon_S}\right) \quad (3)$$

$$E_{CT} = e[E_{OX} - E_{RED}] + \Delta G_S \quad (4)$$

In these equation (equ.2–4), e = electric charge, E_{OX} = half-wave potential for one-electron transfer oxidation of the RB unit, E_{RED} = half-wave potential for one-electron transfer reduction of the NI unit, E_{00} = excitation energy of that particular excited state, ε_S = static dielectric constant of the solvent, ε_0 = permittivity of free space, R_{CC} = center-to-center separation distance determined by DFT calculation studies, R_D = radius of the RB-based donor, R_A =radius of the NI-based acceptor, ε_{REF} = (8.93, DCM) static dielectric constant of the solvent used for the electrochemical studies.[8]

The UV-vis spectroelectrochemical experiment with **RB**-**NI** was performed on a CHI610D electrochemical workstation (Shanghai, China). The electrolytic cell including three electrodes, the Pt gauze auxiliary electrode as working electrode, the platinum disk electrode as counter electrode, and the Ag/$AgNO_3$ (0.1 M in acetonitrile) electrode as reference electrode. According to the redox CV data, the value of first reduction peak was used as the control reduction potential to record the spectroelectrochemical graphs. The measurements were carried out at room temperature in deaerated DCM containing $Bu_4N[PF_6]$ (*ca.* 0.1 M) as supporting electrolyte and at sweep rates of 0.08 mV/s (applied voltage range: 1.72–1.74 eV), and the concentrations of compound **RB**-**NI** was *ca.* 3 × 10^{-3} M.

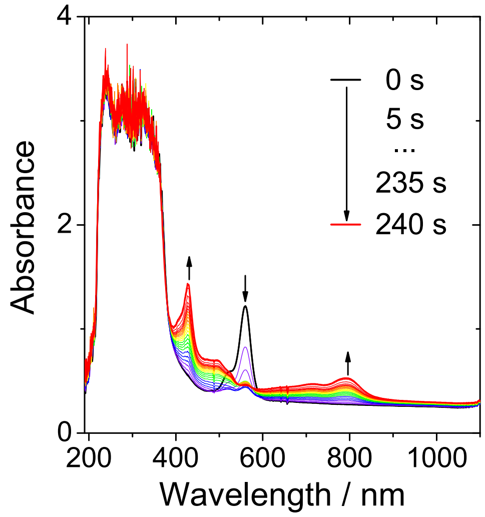

Figure S17. UV–Vis absorption spectra of NI radical anion of **RB-NI**, measured with spectroelectrochemical method in deaerated DCM containing $Bu_4N[PF_6]$ (*ca.* 0.1 M) as supporting electrolyte, Ag/$AgNO_3$ as reference electrode, applied voltage range: 1.72–1.74 eV, $c = 3.0 \times 10^{-3}$ M. 20 °C.

8. Femtosecond Time-resolved Transient Absorption Spectra

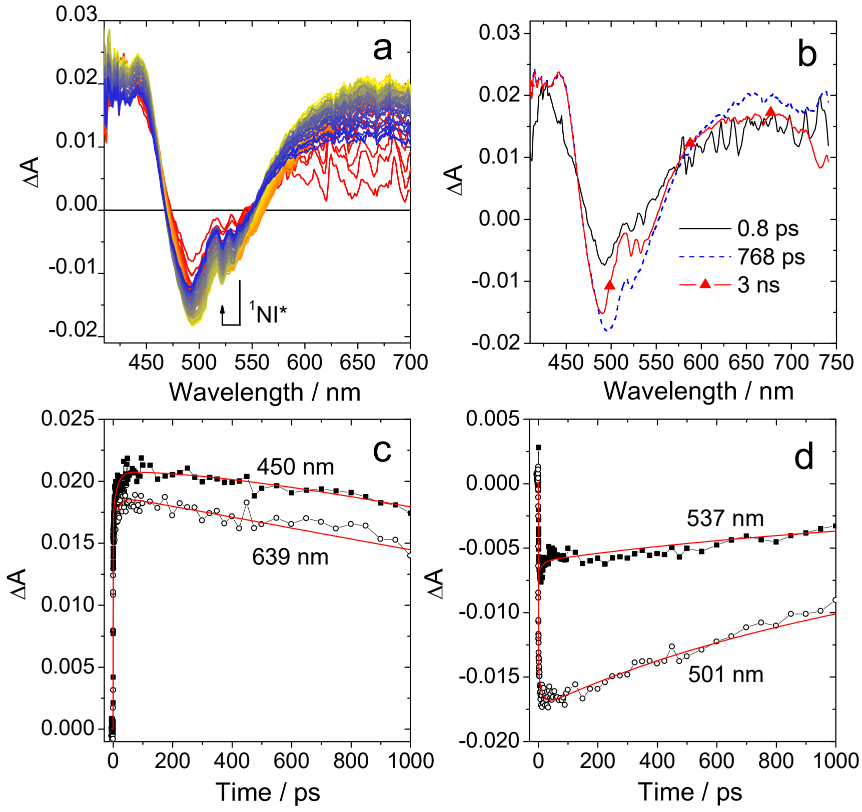

Figure S18. (a) Time resolved transient absorption spectra of **NI-NH$_2$** in toluene, color code goes from red to blue covering the time interval from 0.5 ps to1.5 ns. (b) Evolution associated difference spectra obtained from global analysis of time resolved data in (a) with relative time evolution. Kinetic traces at four selected wavelengths: (c) 450 nm and 639 nm, (d) 537 nm and 501 nm.

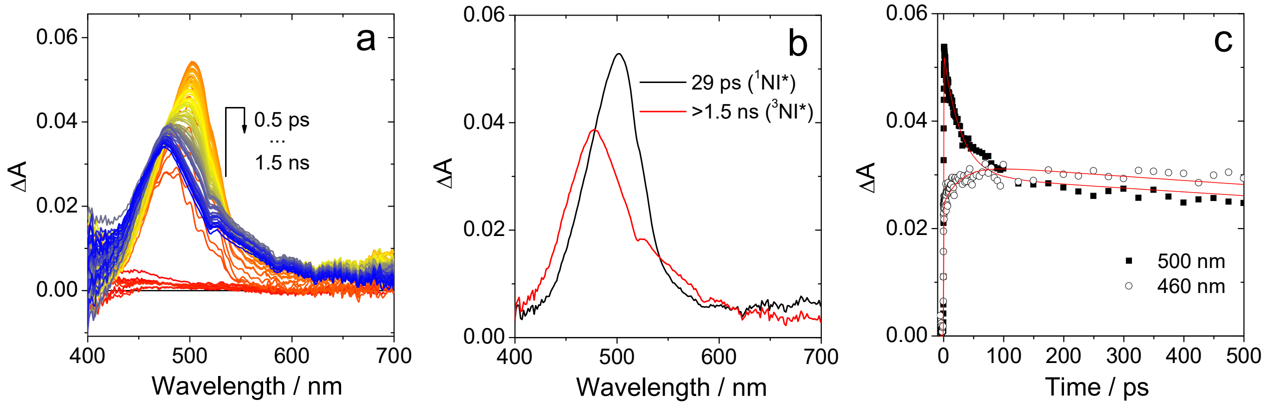

Figure S19. (a) Time resloved transient absorption spectra of **NI-Br** in toluene, color code goes from red to blue covering the time interval from 0.5 ps to1.5 ns. (b) Evolution associated difference spectra obtained from global analysis of time resolved data in (a) with relative time evolution. (c) Selected kinetic traces (symbols) with fit obtained from global analysis (red lines). λ_{ex} = 350 nm.

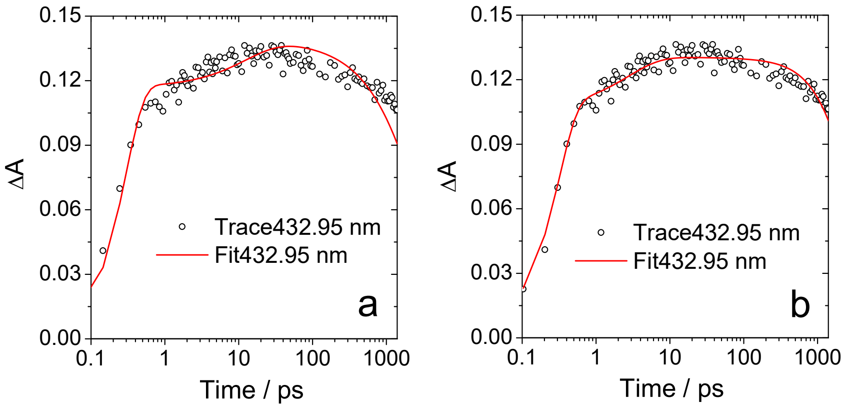

Figure S20. Comparison of the fit obtained with two (a) and three (b) time constants for the time trace at 433 nm measured with transient absorption spectroscopy for RB-NI in toluene. The time axis is on logarithmic scale. λ_{ex} = 400 nm.

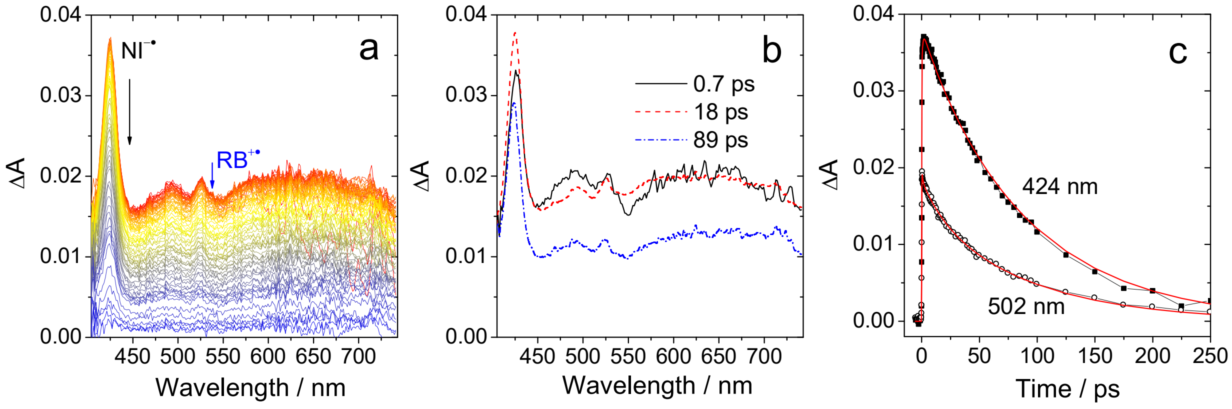

Figure S21. (a) Time resloved transient absorption spectra of **RB-NI** in acetonitrile, color code goes from red to blue covering the time interval from 0.5 ps to1.5 ns. (b) Evolution associated difference spectra obtained from global analysis of time resolved data in (a) with relative time evolution. (c) Selected kinetic traces (symbols) with fit obtained from global analysis (red lines). λ_{ex} = 400 nm.

9. Nanosecond Time-resolved Transient Absorption Spectra

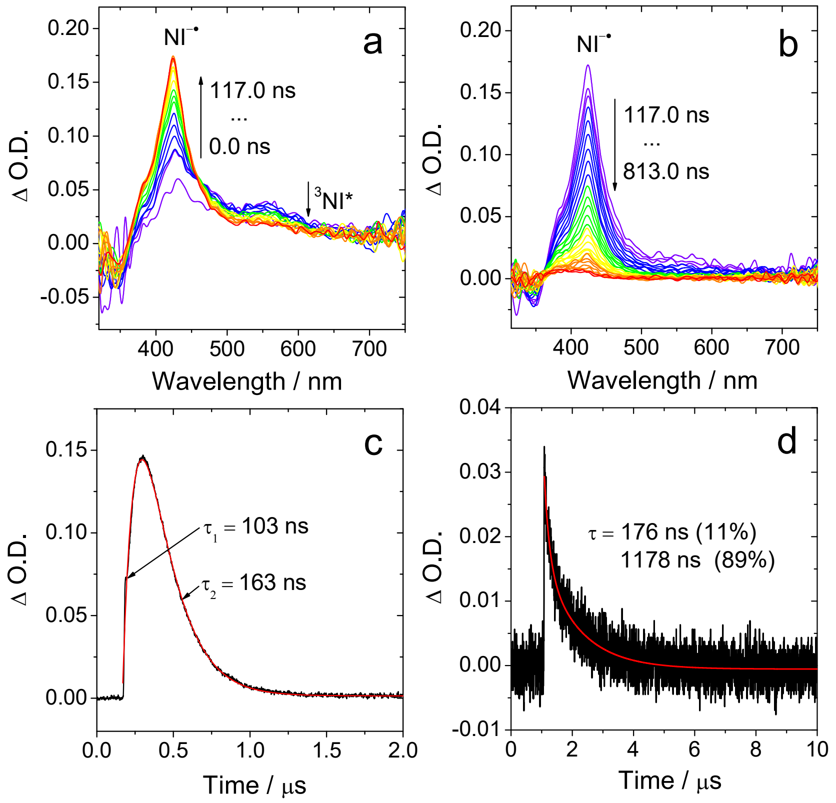

Figure S22. Nanosecond transient absorption spectra of the compound **RB-NI**. (a) Growth of the excited state absorption (ESA) band at 425 nm; (b) the decaying of the ESA band of **RB-NI** at 425 nm; (c) evolution trace of the transient signal at 425 nm; in *n*-hexane. (d) Evolution trace of the transient signal at 540 nm in deaerated *n*-hexane. λ_{ex} = 350 nm, *c* = 4.0 × 10^{-5} M. 20 °C.

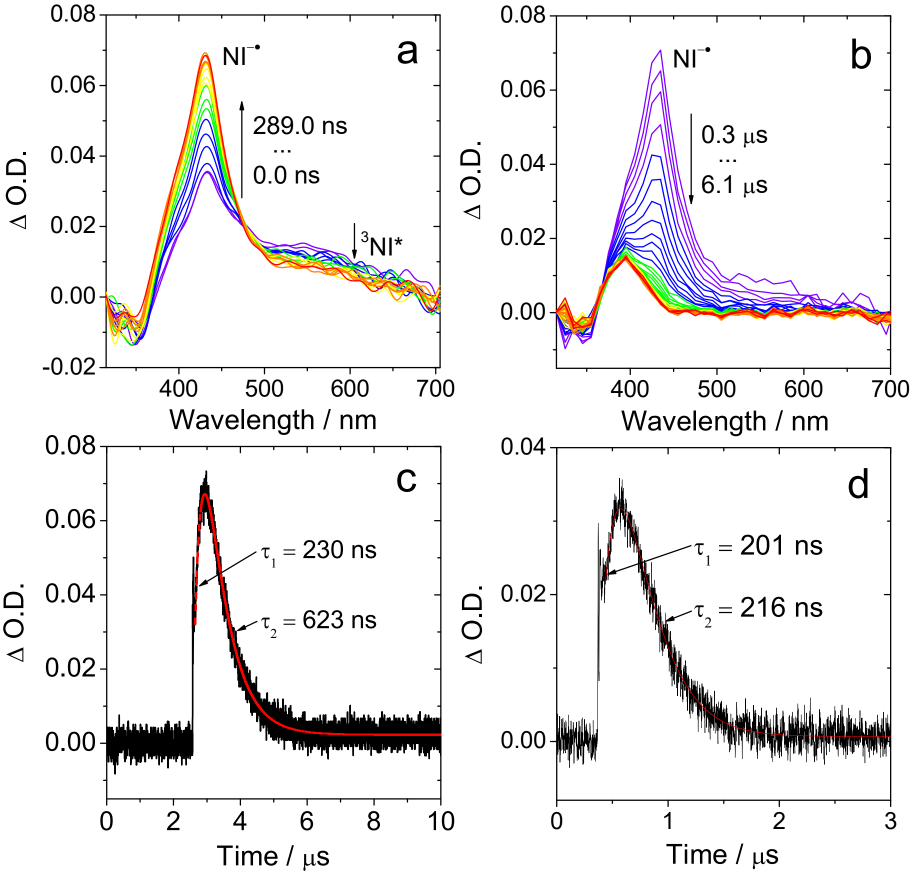

Figure S23. Nanosecond transient absorption spectra of the compound **RB-NI**. (a) Growth of the ESA band at 430 nm; (b) the decaying of the ESA band of **RB-NI** at 430 nm; (c) evolution trace of the transient signal at 435 nm; in deaerated toluene. (d) Evolution trace of the transient signal at 435 nm; in toluene. λ_{ex} = 350 nm, c = 2.0 × 10^{-5} M, 20 °C.

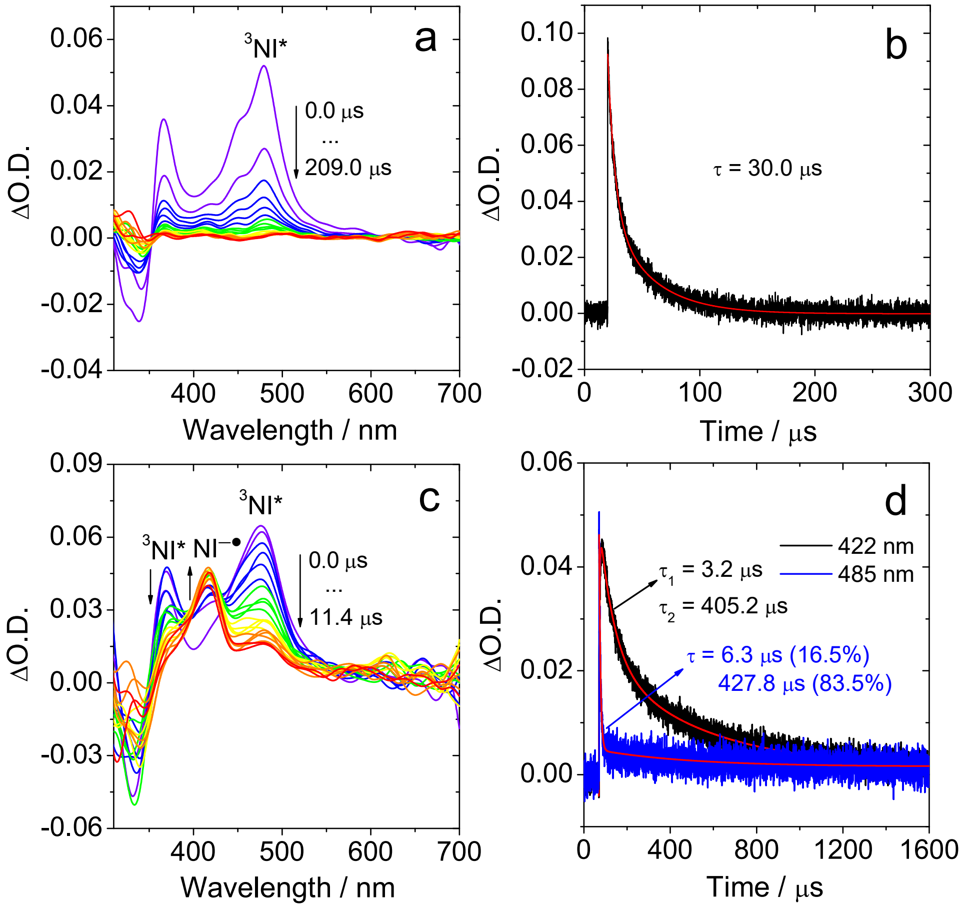

Figure S24. Transient absorption spectra of **NI-Br** (a) in the absence and (c) in the presence of triethylamine (TEA, 250 equiv.) as electron donor. The corresponding decay traces of triplet state of **NI-Br**, (b) without TEA and (d) with TEA (250 equiv.) added as electron donor. λ_{ex} = 355 nm, in deaerated *n*-hexane, $c_{\text{[NI-Br]}} = 2.0 \times 10^{-5}$ M (without TEA), $c = 2.0 \times 10^{-5}$ M (with TEA), 20 °C.

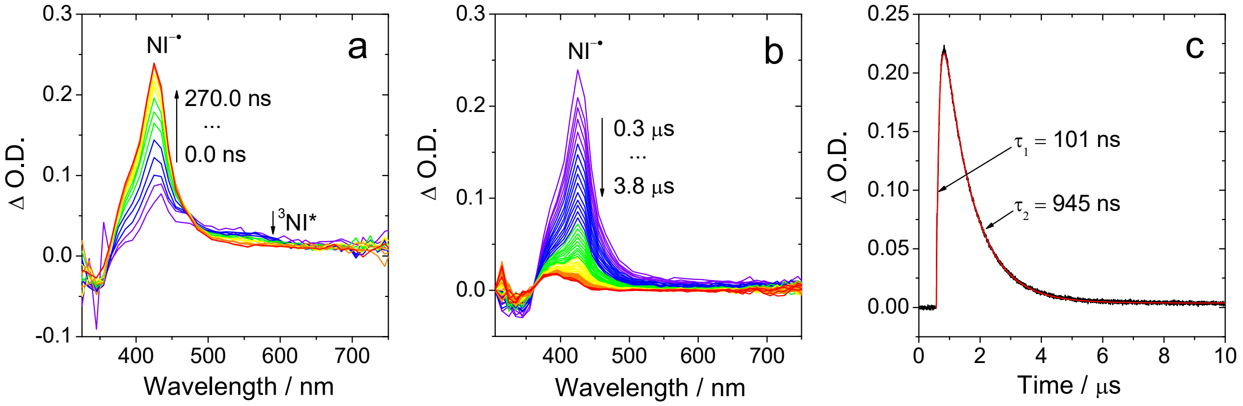

Figure S25. Nanosecond transient absorption spectra of the compound **RB-NI**. (a) Growth of the ESA band of **RB-NI**; (b) the decaying of the ESA band of **RB-NI** at 425 nm; (c) evolution trace of the transient signal at 425 nm; in deaerated polydimethylsiloxane (PDMS), λ_{ex} = 350 nm, c = 4.0 × 10^{-5} M. 20 °C.

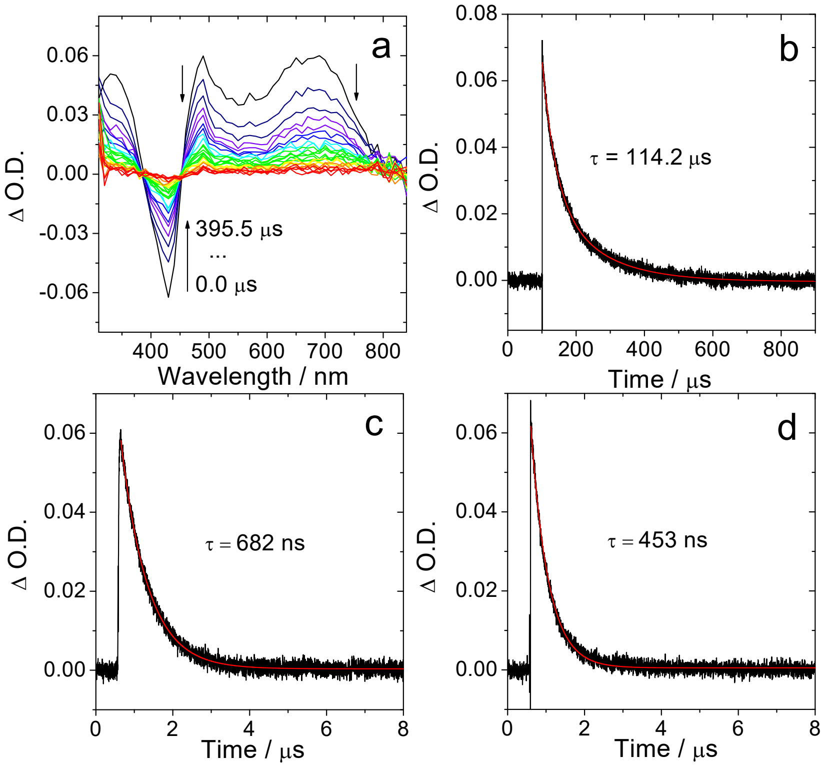

Figure S26. Nanosecond transient absorption spectra of the compound **RB-NI-N**. (a) Transient absorption spectrum of **RB-NI-N**; evolution trace of the transient signal of **RB-NI-N** at 485nm, (b) in deaerated toluene, (c) in *n*-hexane and (d) in toluene; λ_{ex} = 427 nm, c = 5.0 × 10^{-5} M. 20 °C.

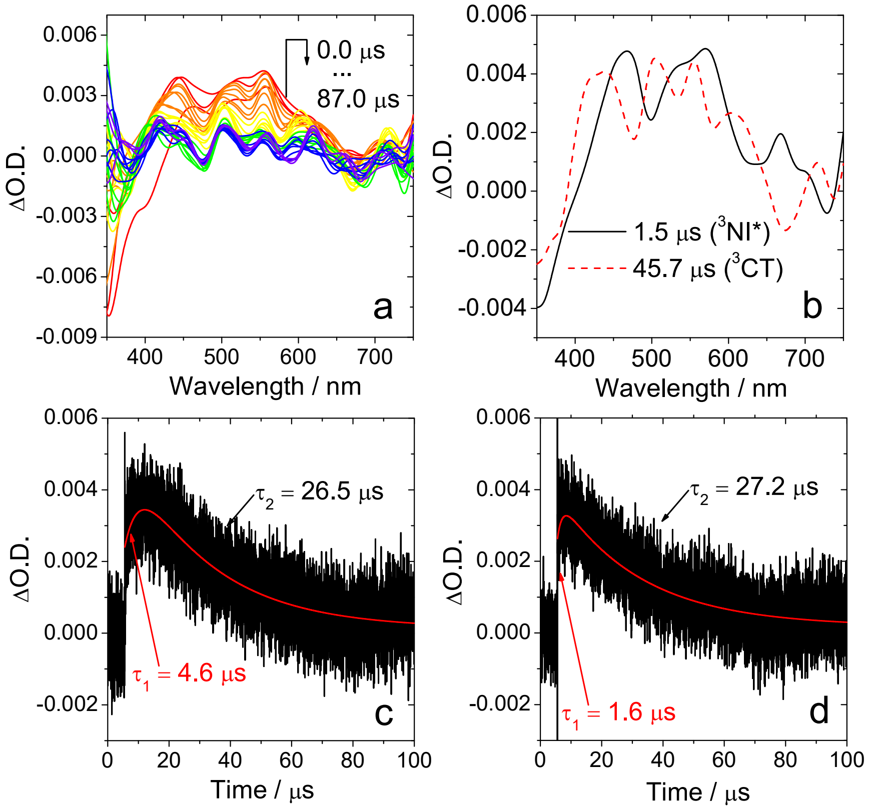

Figure S27. Nanosecond transient absorption spectra of the compound **RB-NI** at 177 K: (a) the ESA spectrum of **RB-NI** at 425 nm and (b) EADS spectra obtained from global analysis of time resolved data in (a) with relative time evolution, evolution trace of the transient signal at (c) 425 nm and (d) 540 nm. In deaerated *n*-hexane (freezing point: 178 K), λ_{ex} = 355 nm, *c* = 4.0 × 10^{-5} M. 20 °C.

In Figure S27, evolution traces of **RB-NI** show growth and decay at 425 nm and 540 nm (at 177 K). EADS spectra of it were obtained from global analysis show the initial state (black solid line) is ^{3}NI* state and the final long-lived state (red dotted line) is ^{3}CT.

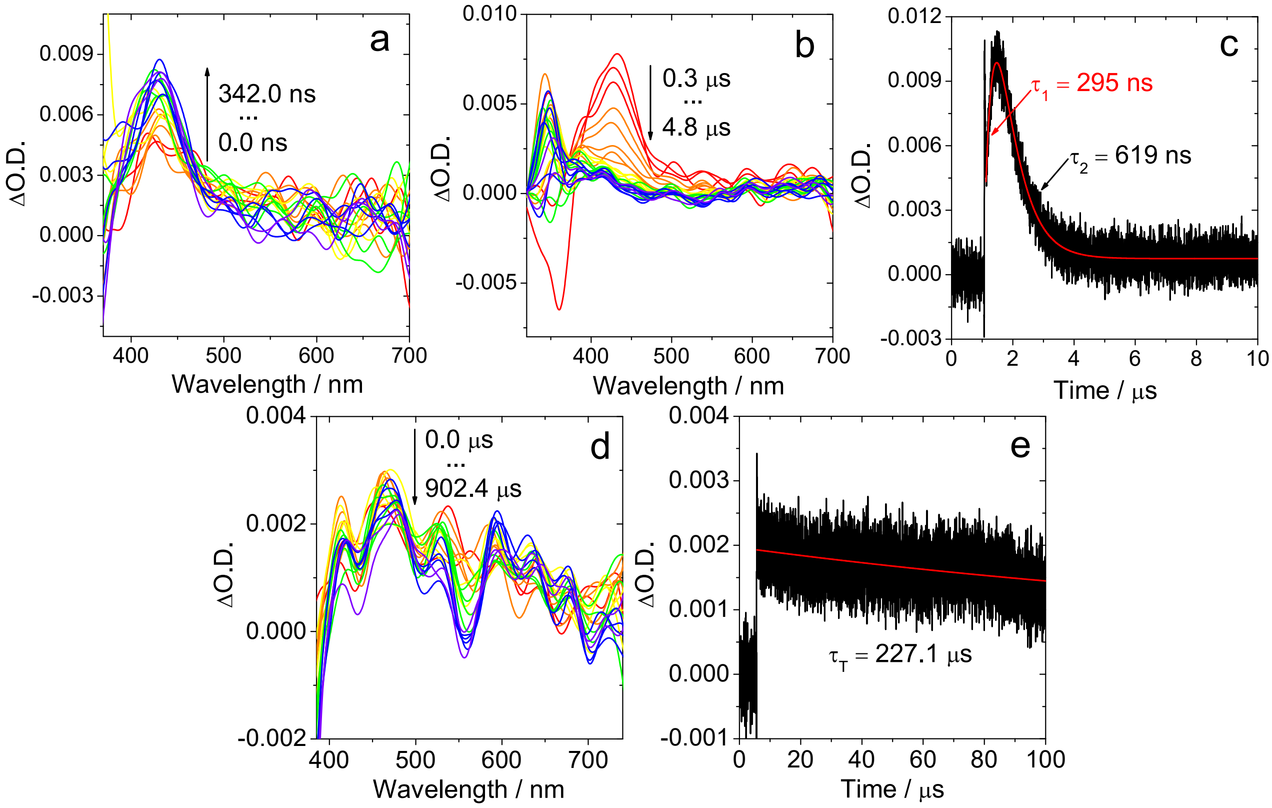

Figure S28. Nanosecond transient absorption spectra of the compound **RB-NI** at 293 K: (a) growth of the ESA band at 425 nm, (b) the decaying of the ESA band at 425 nm, and (c) evolution trace of the transient signal at 425 nm. Nanosecond transient absorption spectra of the compound **RB-NI** at 80 K: (d) the ESA spectrum of **RB-NI** and (e) evolution trace of the transient signal at 425 nm. In deaerated toluene/MeTHF = 3:1 (v/v), λ_{ex} = 355 nm, c = 4.0 × 10^{-5} M.

Table S6. Triplet lifetimes of transient absorption observed for the excitation of **RB-NI**, **RB-NI-N** and **NI-Br**.

Compound	Solvent	Wavelength / nm	Time
**RB-NI**[a]	*n*-hexane	425	125 ns (raise)<br>935 ns (decay)
	Toluene	435	230 ns (raise)<br>623 ns (decay)
	DCM	425	[e]
	MeCN	425	[e]
**RB-NI-N**[b]	*n*-hexane	485	50.5 μs
	Toluene	485	114.2 μs
	DCM	485	136.0 μs
	MeCN	485	[e]
**NI-Br**	*n*-hexane[c]	485	98.2 μs
	Toluene[d]	485	108.1 μs
	DCM[c]	485	159.2 μs
	MeCN[c]	485	119.2 μs

[a] $\lambda_{ex} = 350$ nm, $c = 4.0 \times 10^{-5}$ M in *n*-hexane, $c = 2.0 \times 10^{-5}$ M in toluene, 20 °C. [b] $\lambda_{ex} = 427$ nm, $c = 5.0 \times 10^{-5}$ M, 20 °C. [c] $\lambda_{ex} = 355$ nm, $c = 1.0 \times 10^{-5}$ M, collinear mode, 20 °C. [d] $\lambda_{ex} = 355$ nm, $c = 5.0 \times 10^{-5}$ M, 20 °C. [e] Not observed.

10. Time-resolved Electron Paramagnetic Resonance Spectral Data

Table S7. Zero-Field Splitting Parameters (*D* and *E*) [a] and Relative Populations $p_{x,y,z}$ [a] of the Spin States at Zero Magnetic Field of the Compounds.

Molecule	*D* / mT	*E* / mT	p_x	p_y	p_z
**NI-Br**	90.2	4.6	0.357	0.643	0
**RB-NI**	84.9	2.8	0.828	0.172	0
**RB-NI-N**	62.2	3.6	0.472	0.528	0

[a] Obtained from the simulations of the triplet state TR-EPR spectra in a toluene/MeTHF matrix at 80 K. Note: For the assignment of p_1, p_2 and p_3, the convention outlined in was used, i.e., D > 0 and E > 0 for an oblate spin-density distribution.[9] An isotropic *g*-value of 2.002 was chosen for the spectral simulations of all three molecules. The *D* and *E* values are accurate within 0.2 mT. Different gaussian and Lorentzian linewidth were used for the simulations.

11. Excited State Energy Diagram of RB-NI-N

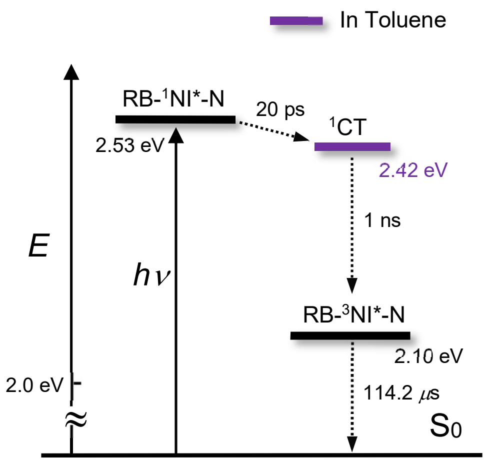

Scheme S2. Simplified Jablonski Diagram Illustrating the Photophysical Processes Involved in (a) RB-NI and (b) RB-NI-N.

[a] ^{1}CT energy levels were calculated based on the electrochemical data and TDDFT computations. TDDFT calculations are performed at the B3LYP/6–31G(d) level by using Gaussian 09W. The triplet excited state energy levels of **RB-NI** and **RB-NI-N** are obtained from phosphorescence at 77 K.

References

[1] a) P. Zhou, J. Yao, G. Hu, J. Fang, *ACS Chem. Biol.* **2016**, *11*, 1098–1105; b) P. Mahato, S. Saha, E. Suresh, R. Di Liddo, P. P. Parnigotto, M. T. Conconi, M. K. Kesharwani, B. Ganguly, A. Das, *Inorg. Chem.* **2012**, *51*, 1769–1777; c) X.-L. Liu, X.-J. Du, C.-G. Dai, Q.-H. Song. *J. Org. Chem.* **2014**, *79*, 9481–9489; d) W.-Y. Liu, H.-Y. Li, B.-X. Zhao, J.-Y. Miao. *Org. Biomol. Chem.* **2011**, *9*, 4802–4805; e) M. Dong, Y.-W. Wang, Y. Peng. *Org. Lett.* **2010**, *12*, 5310–5313.

[2] E. R. Henry, J. Hofrichter. In *Methods in Enzymology*, **1992**, *210*, 129–192.

[3] J. J. Snellenburg, S. Laptenok, R. Seger, K. M. Mullen, I. H. M. van Stokkum. *J. Stat. Software* **2012**, *49*, 1–22.

[4] Aly, S. M.; Ahmed, G. H.; Shaheen, B. S.; Sun, J.; Mohammed, O. F. *J. Phys. Chem. Lett.* **2015**, *6*, 791–795.

[5] (a) G. M. Sheldrick. *Acta Cryst.* **2015**, *C71*, 3–8. (b) G. M. Sheldrick. *Acta Cryst*. **2015**, *A71*, 3–8. (c) O. V. Dolomanov, L.J. Bourhis, R.J Gildea, J. A. K. Howard and H. Puschmann. *J. Appl. Cryst.* **2009**, *42*, 339–341.

[6] a) A. D. Becke, *J. Chem. Phys.* **1993,** *98*, 5648–5652; b) M. J. Frisch, G. W. Trucks, H. B. Schlegel, G. E. Scuseria, M. A. Robb, J. R. Cheeseman, G. Scalmani, V. Barone, B. Mennucci, G. A. Petersson, et al. Gaussian 09, revision A.1; Gaussian, Inc.: Wallingford, CT, **2009**.

[7] A. A. Abdel-Shafi, P. D. Beer, R. J. Mortimer, F. Wilkinson, *Phys. Chem. Chem. Phys.* **2000**, *2*, 3137–3144.

[8] R. Ziessel, B. D. Allen, D. B. Rewinska, A. Harriman, *Chem. Eur. J.* **2009,** *15*, 7382–7393.

[9] S. Richert, C. E. Tait, C. R. Timmel, *J. Magn. Reson.* **2017**, *280*, 103–116.
